# Supplementary material for: Ligustilide Inhibits Tumor Angiogenesis by Downregulating VEGFA Secretion from Cancer-Associated Fibroblasts in Prostate Cancer via TLR4
Source: Cancers (Basel). 2022 May 13;14(10):2406. doi: 10.3390/cancers14102406 (PMC9140166; doi:10.3390/cancers14102406)
Supplement: Supplementary file 1 [file cancers-14-02406-s001.zip › Figures S1-S6.pdf]

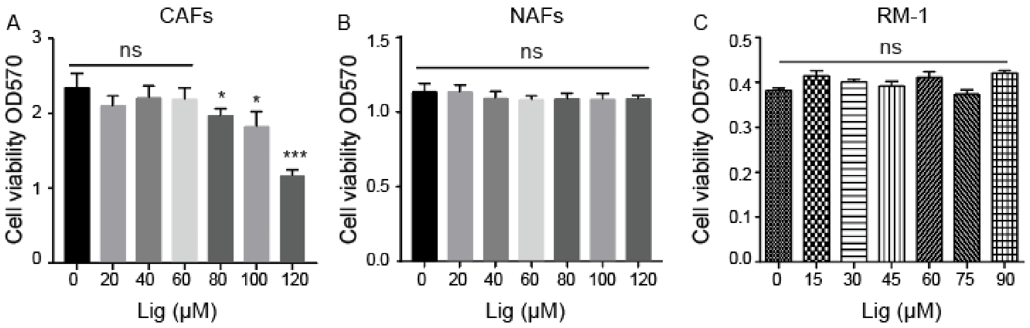

Figure S1. Ligustilide had no influence on the proliferation of CAFs, NAFs and RM-1 cells. Ligustilide had no effect on the proliferation of CAFs at concentrations of 0, 20, 40 and 60 μM for 48 h (A). Ligustilide had no influence on the proliferation of NAFs and RM-1 cells for 24 h at different concentrations (B-C). **p*<0.05 and ****p*< 0.001, ns: not significant.

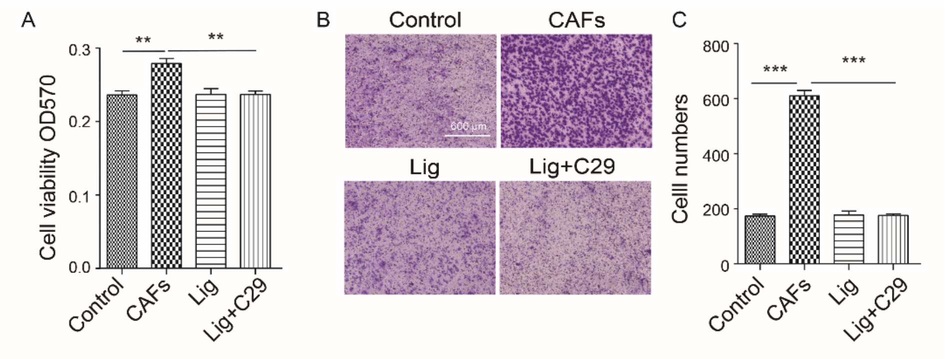

Figure S2. Ligustilide did not act via the TLR2 in CAFs. C29 (TLR2 inhibitor 100 μM) had no influence on the inhibitory effect of ligustilide-treated CAF supernatant on the proliferation (A) or migration (B-C) of HUVECs. ***p*< 0.01 and ****p* < 0.001.

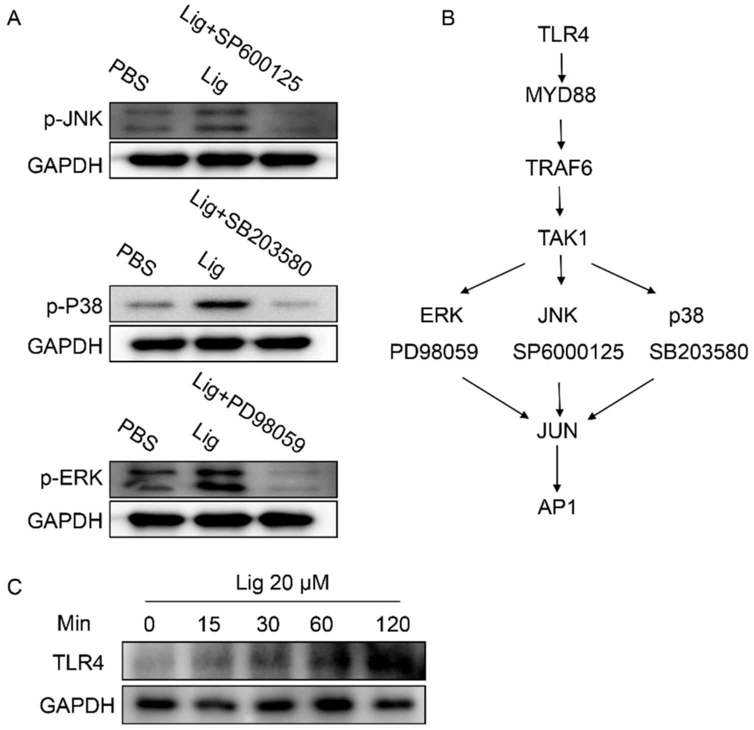

Figure S3. Ligustilide activated AP-1 by activating p-ERK, p-p38, p-JNK and TLR4 in CAFs. Upregulation of p-ERK, p-p38 and p-JNK induced with ligustilide was blocked by PD98059, SB203580 and

SP600125, respectively, in CAFs (A). Ligustilide/TLR4/ERK, JNK, p38/AP1 axis (B). Upregulation of TLR4 was induced by ligustilide in CAFs (C). The original blots could be found in Figure S7.

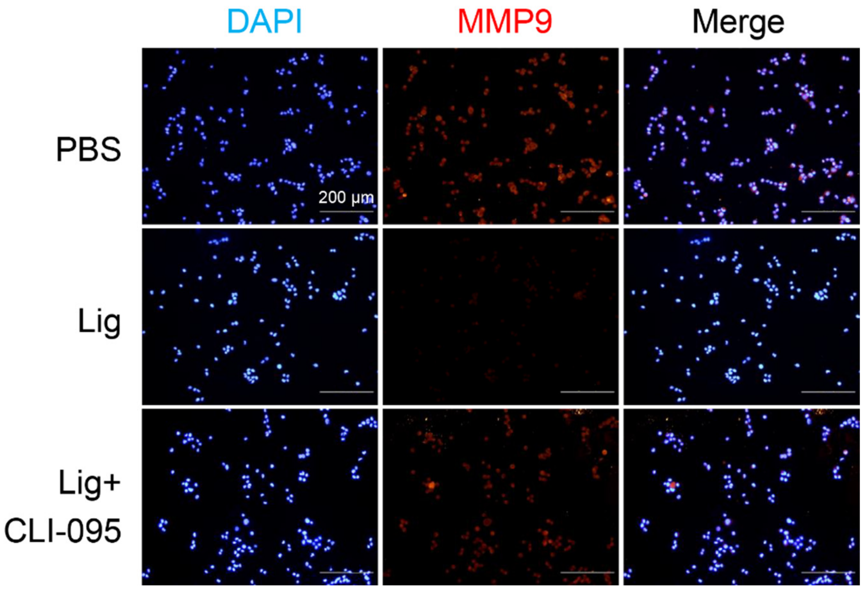

Figure S4. Ligustilide inhibited MMP9 expression in CAFs via TLR4.

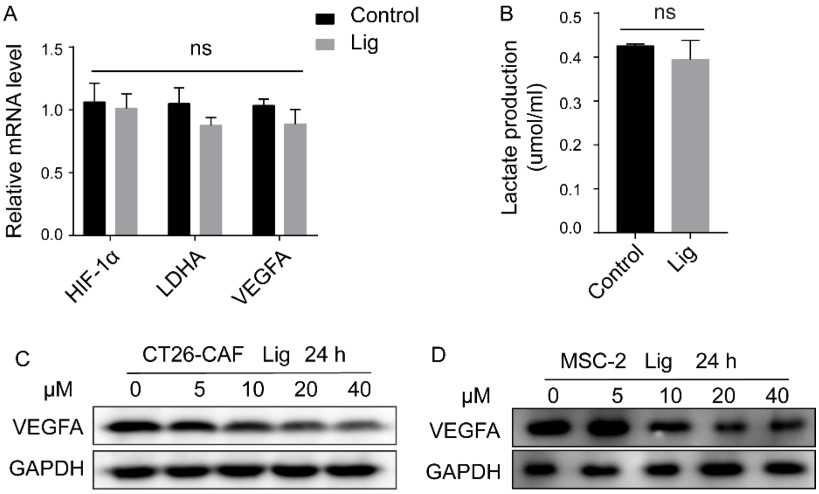

Figure S5. Ligustilide did not affect the expression of HIF-1α/VEGFA or glycolysis in NAFs but down-regulated VEGFA both in CT26-CAF[1] and myeloid derived suppressor cell line (MSC-2)[2]. Ligustilide did not affect HIF-1α, VEGFA, or LDHA at the mRNA level in NAFs (A). Ligustilide did not affect lactate production in NAFs (B). Ligustilide down-regulated VEGFA in CT26-CAF (C) and MSC-2(D)with time dependent effect. The original blots could be found in Figure S7.

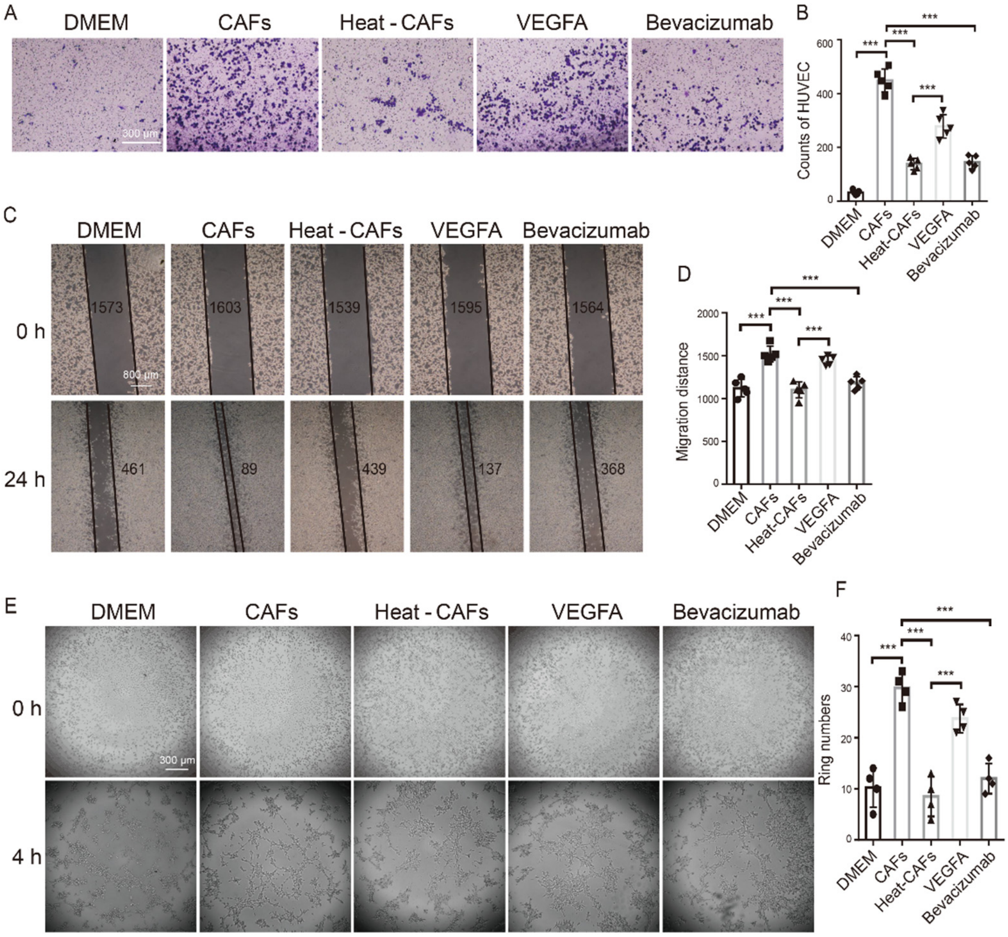

Figure S6. The effect of ligustilide on CAFs promotion to angiogenesis can be blocked by heating and VEGFA antibody (Bevacizumab). CAF supernatant was pretreated by heating (100 ℃, 5min) , heating and adding VEGFA, and VEGFA monoclonal antibody (Bevacizumab), respectively. The effect of CAF supernatant on HUVEC migration in Transwell culture systems and statistical results of HUVECs that migrated into the lower chamber of the Transwell (A,B). Migration in scratch assay and statistical results (C, D). Tube-like structure formation and statistical results (E, F). *** $p < 0.001$.
