# Supplementary figures and images for: Ligustilide Inhibits Tumor Angiogenesis by Downregulating VEGFA Secretion from Cancer-Associated Fibroblasts in Prostate Cancer via TLR4
Source: Cancers (Basel). 2022 May 13;14(10):2406. doi: 10.3390/cancers14102406 (PMC9140166; doi:10.3390/cancers14102406)

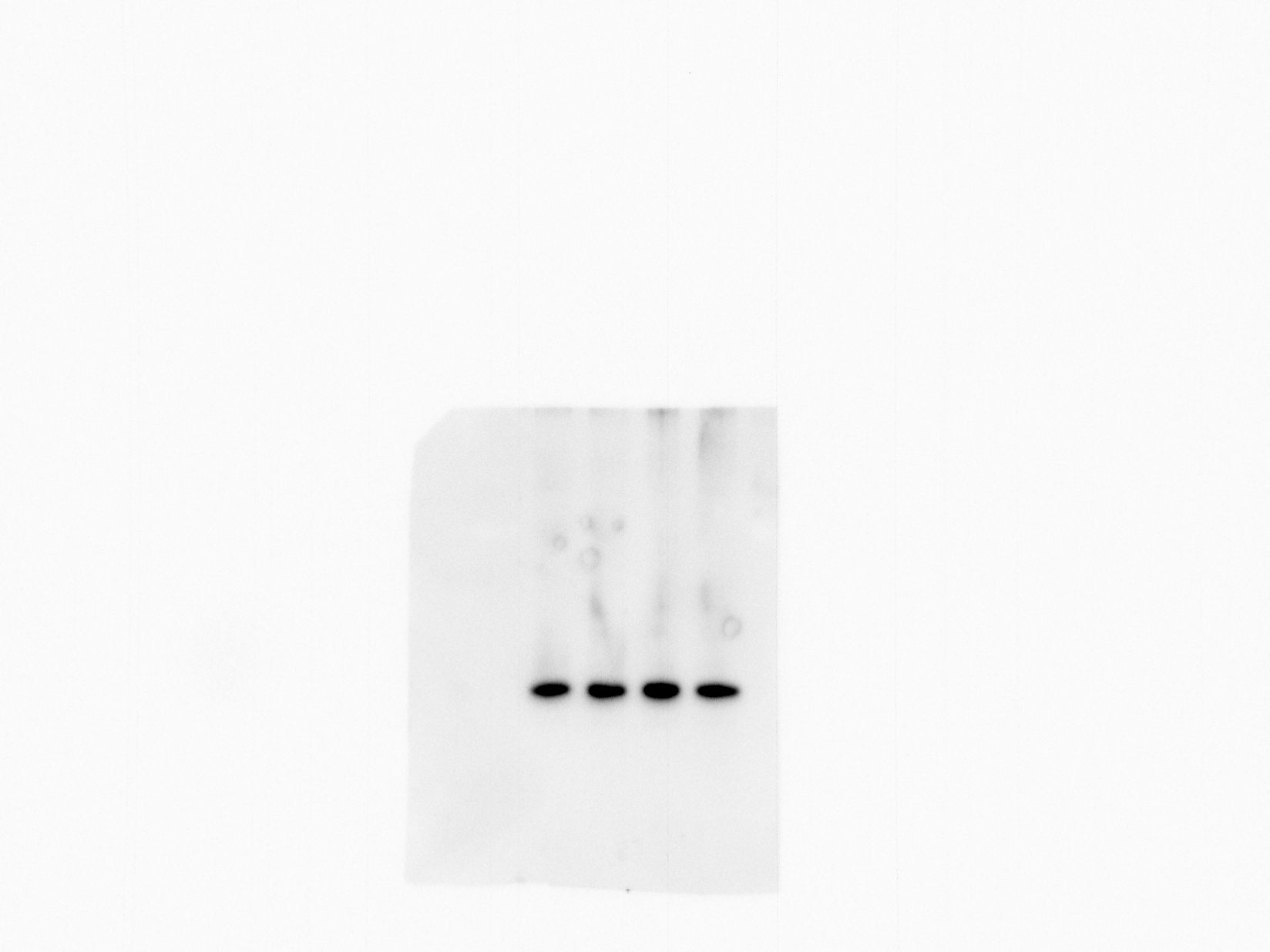

Supplement: Supplementary file 1 [file cancers-14-02406-s001.zip › Figure S7 original blots/Fig3G-gapdh-1.jpg]

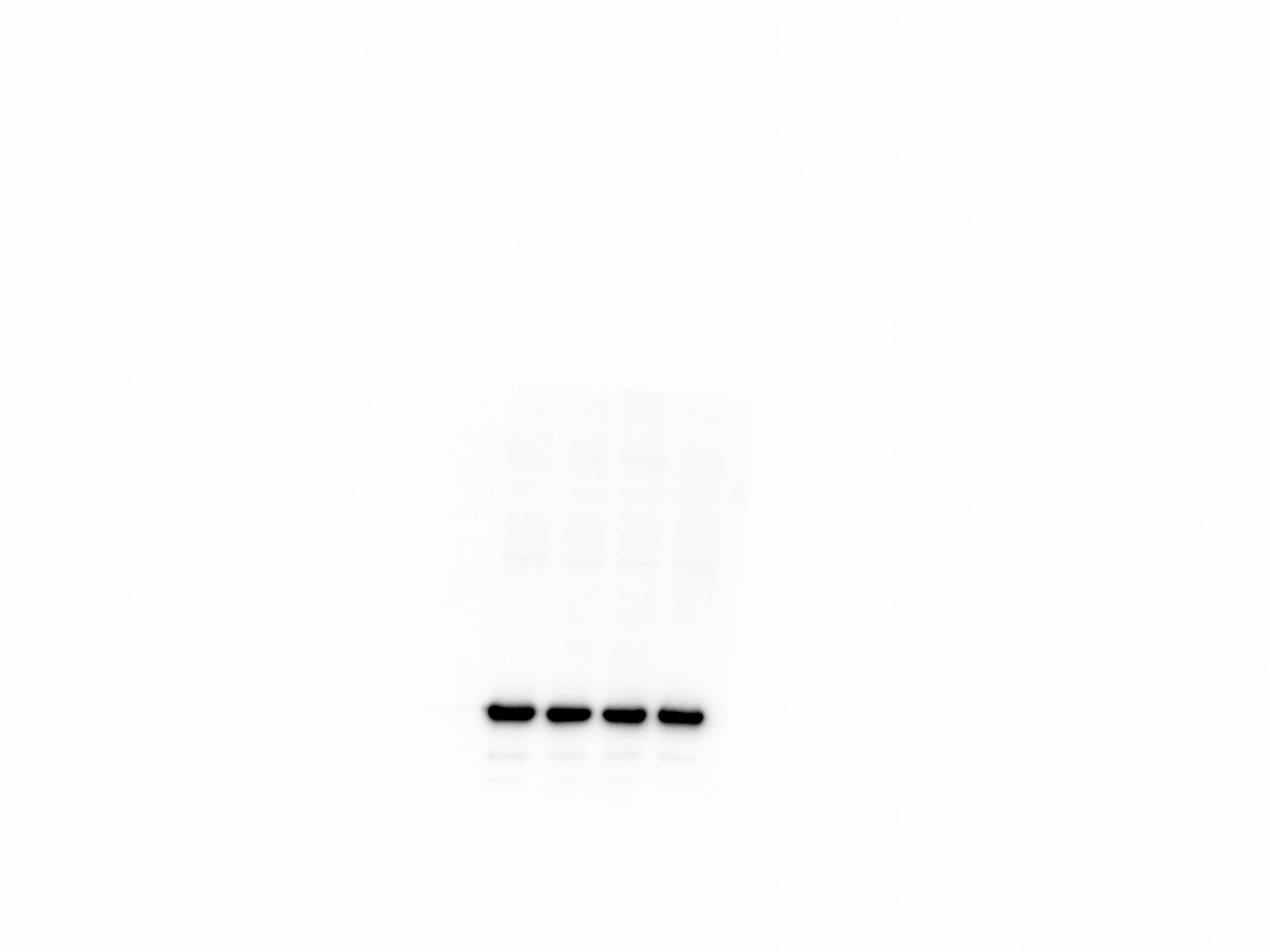

Supplement: Supplementary file 1 [file cancers-14-02406-s001.zip › Figure S7 original blots/Fig3G-gapdh-2.jpg]

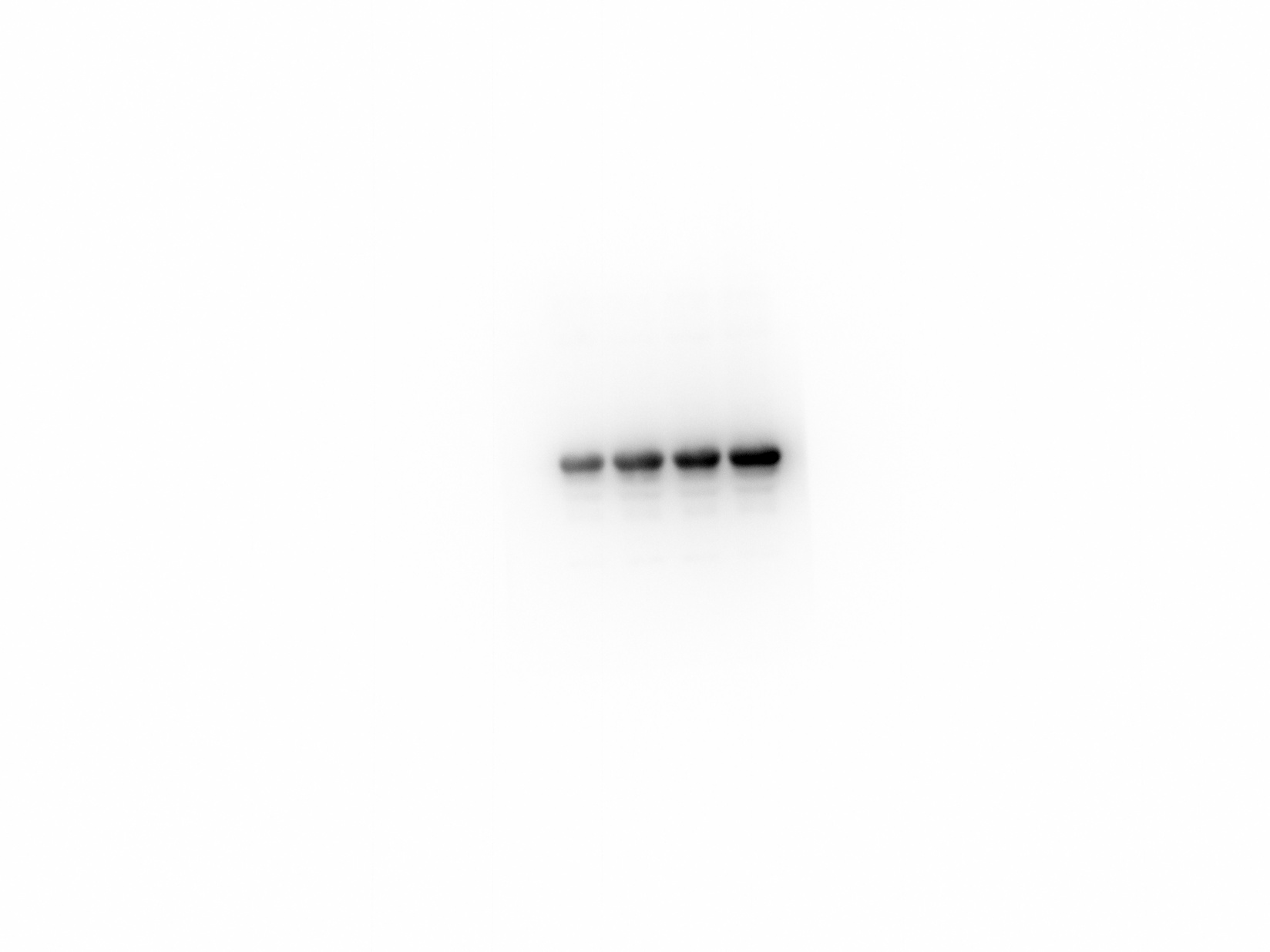

Supplement: Supplementary file 1 [file cancers-14-02406-s001.zip › Figure S7 original blots/Fig3G-myd88-1.jpg]

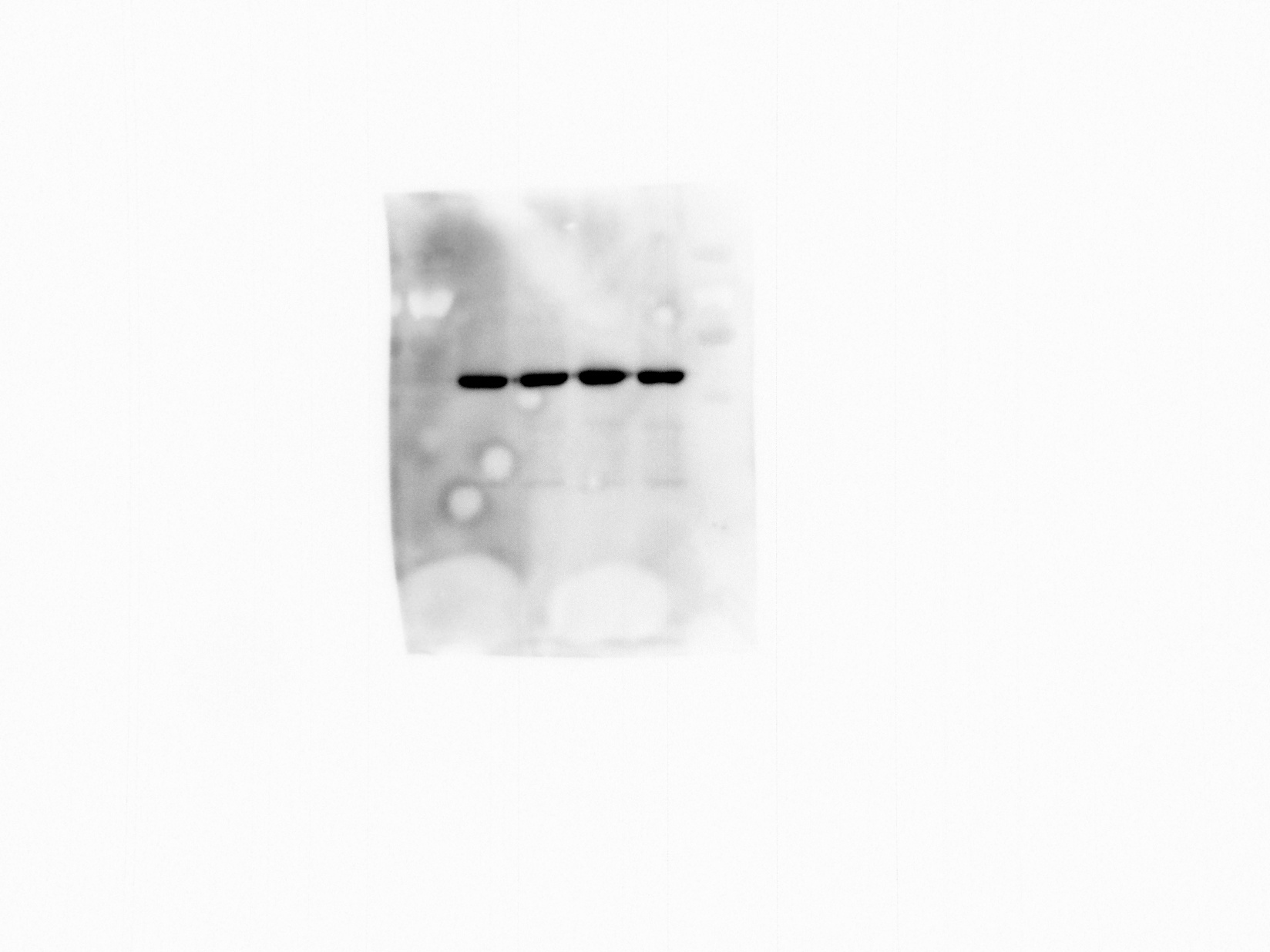

Supplement: Supplementary file 1 [file cancers-14-02406-s001.zip › Figure S7 original blots/Fig3G-myd88-2.jpg]

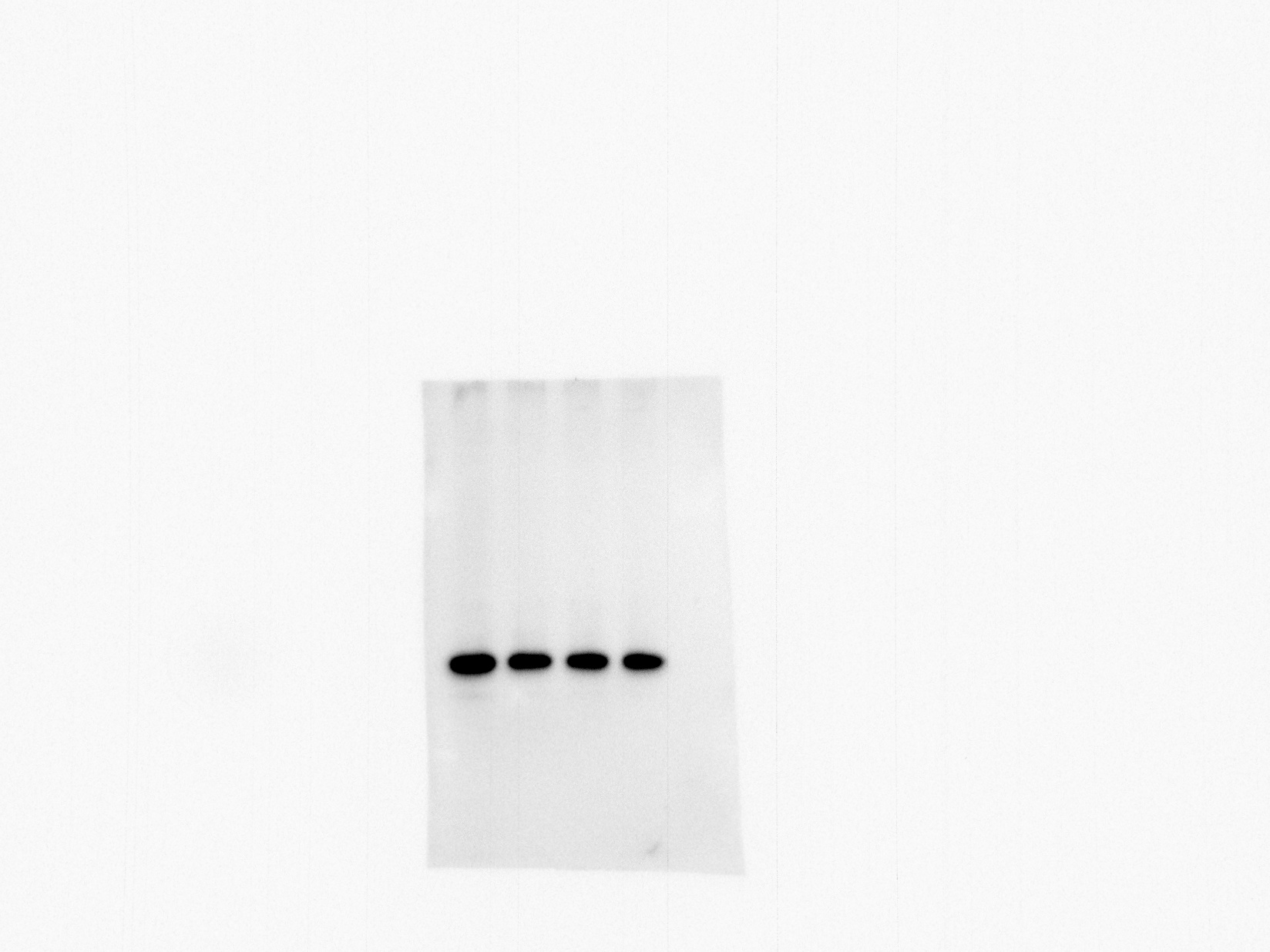

Supplement: Supplementary file 1 [file cancers-14-02406-s001.zip › Figure S7 original blots/Fig3G-P38-1.jpg]

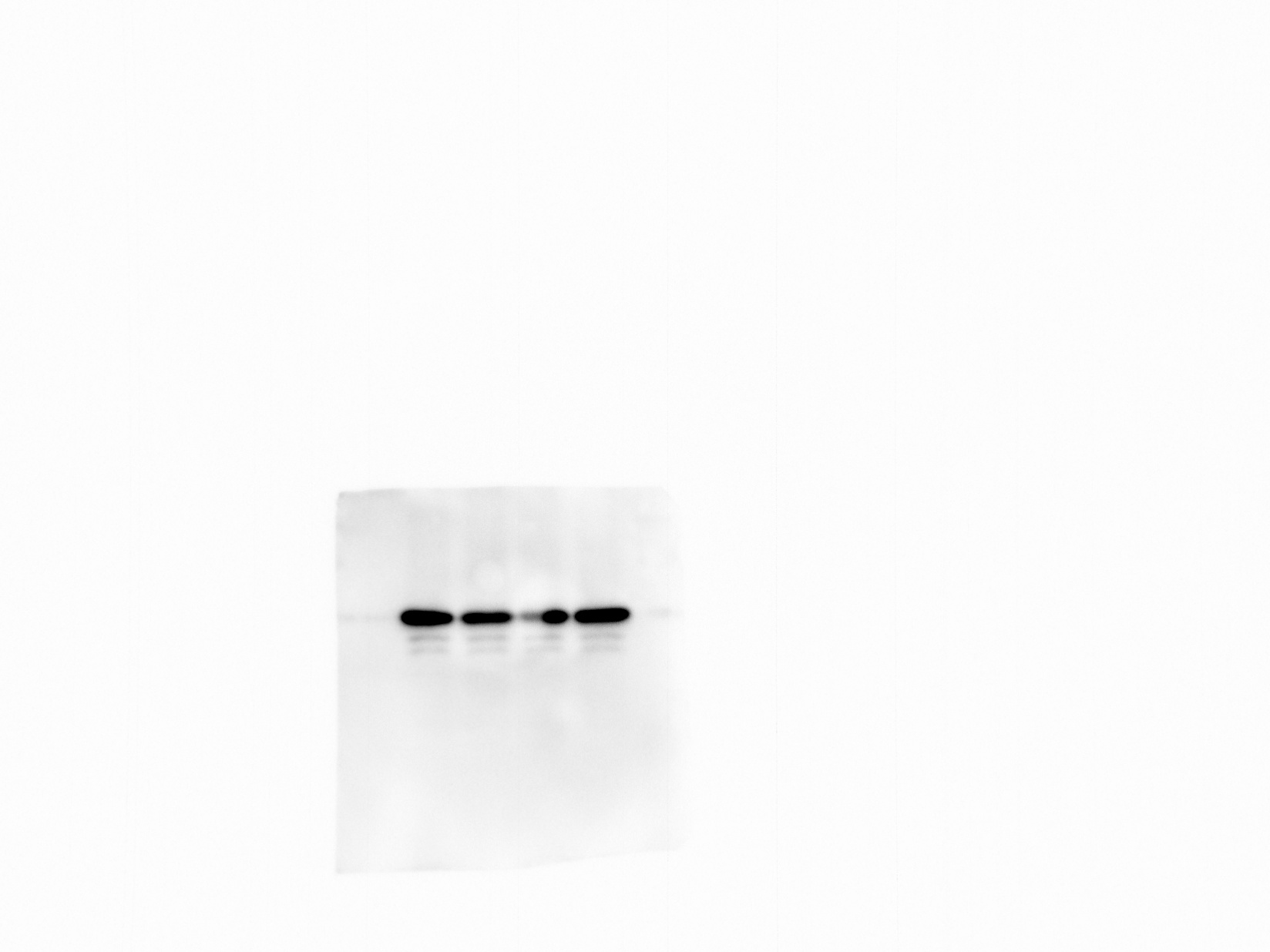

Supplement: Supplementary file 1 [file cancers-14-02406-s001.zip › Figure S7 original blots/Fig3G-P38-2.jpg]

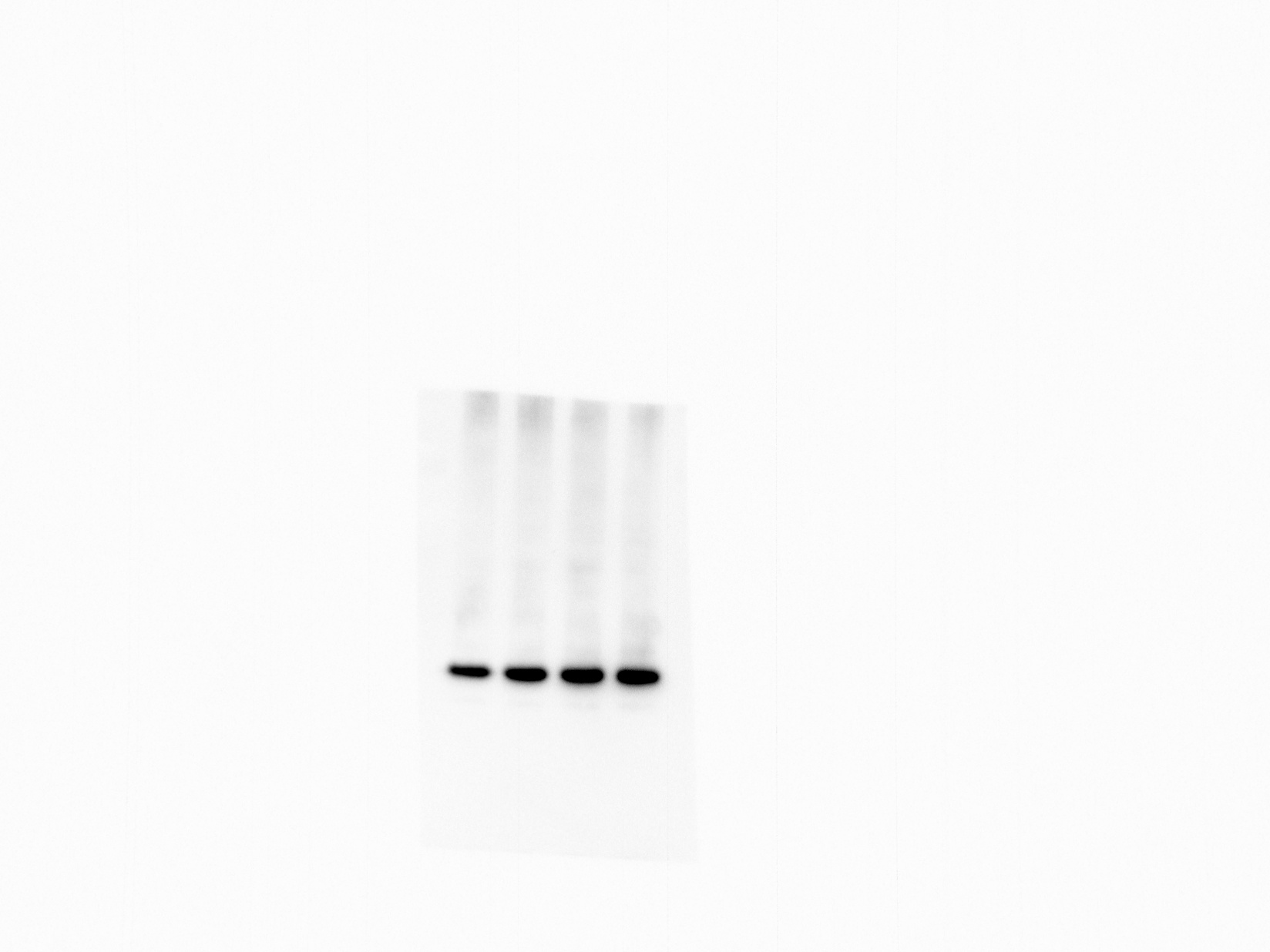

Supplement: Supplementary file 1 [file cancers-14-02406-s001.zip › Figure S7 original blots/Fig3G-PP38-1.jpg]

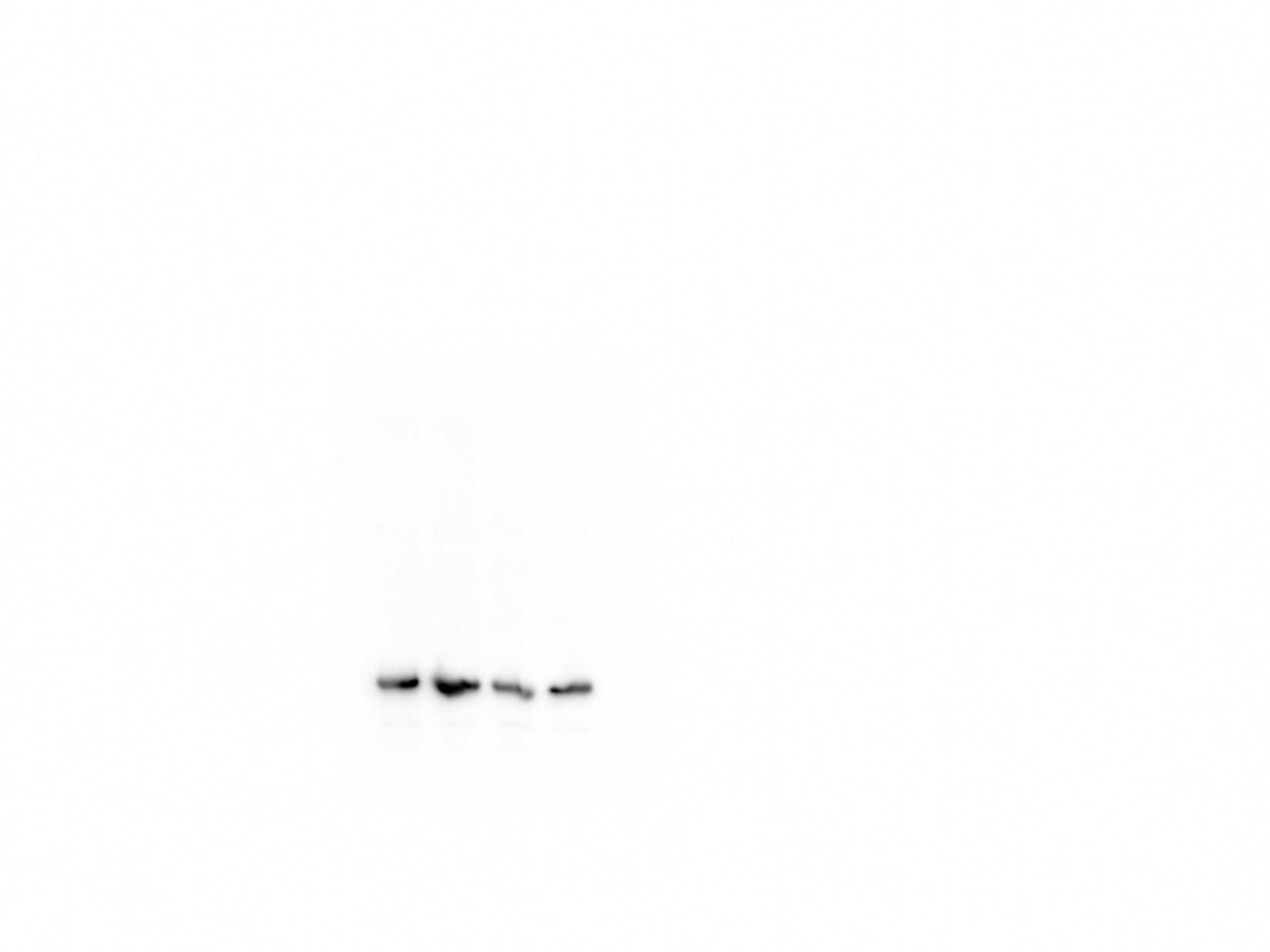

Supplement: Supplementary file 1 [file cancers-14-02406-s001.zip › Figure S7 original blots/Fig3G-pp38-2.jpg]

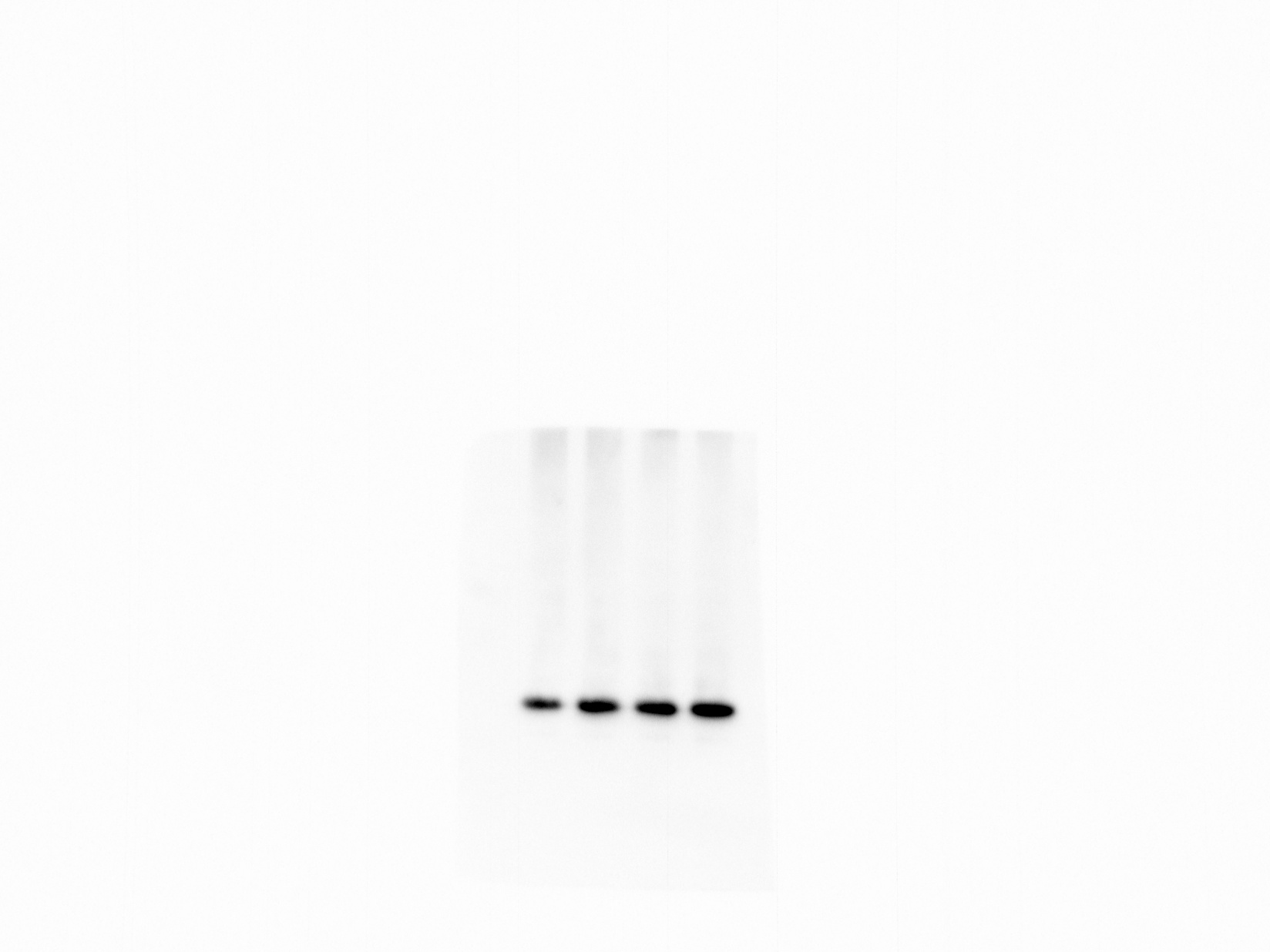

Supplement: Supplementary file 1 [file cancers-14-02406-s001.zip › Figure S7 original blots/Fig3G-PTAK1-1.jpg]

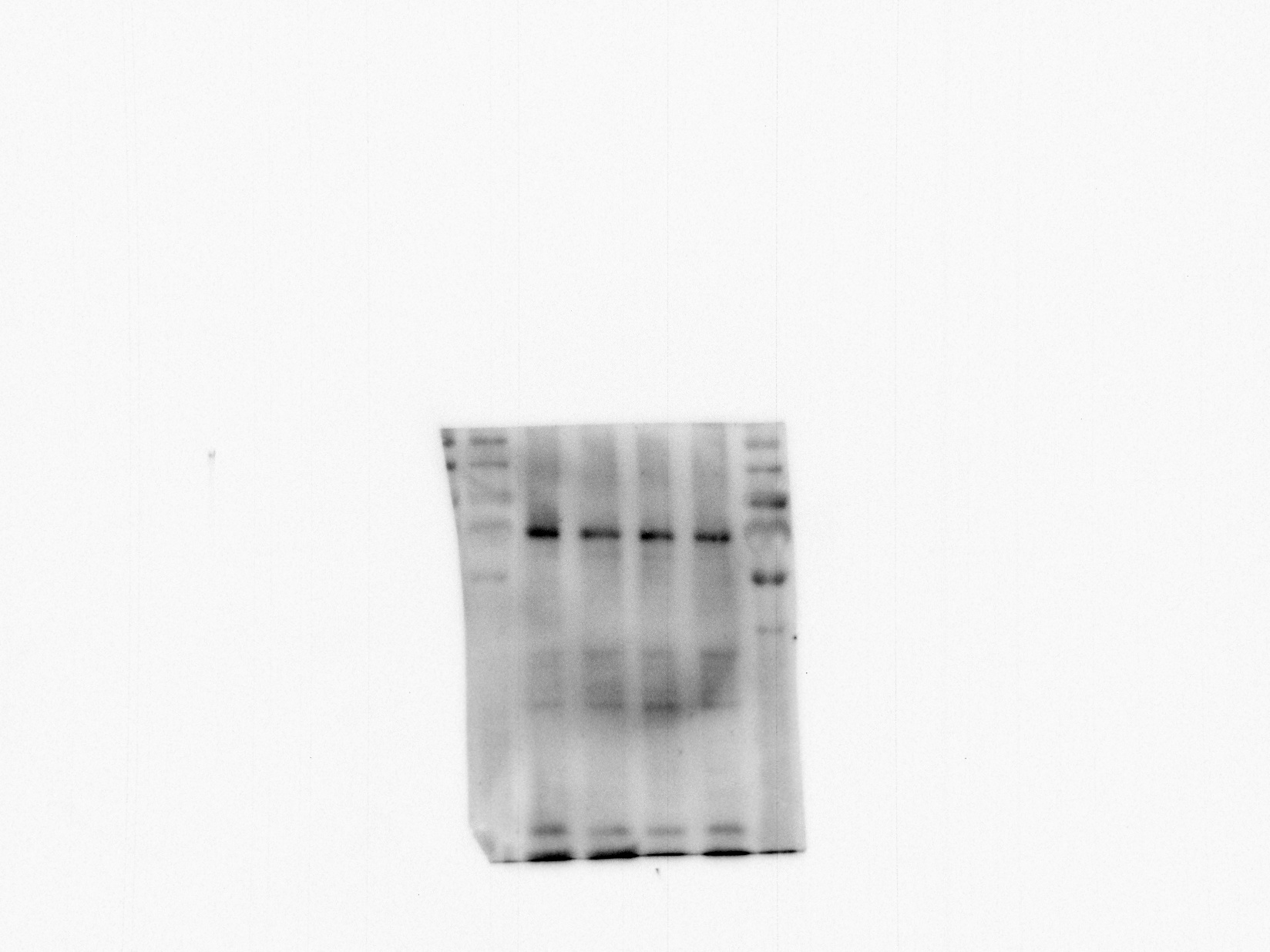

Supplement: Supplementary file 1 [file cancers-14-02406-s001.zip › Figure S7 original blots/Fig3G-ptak1-2.jpg]

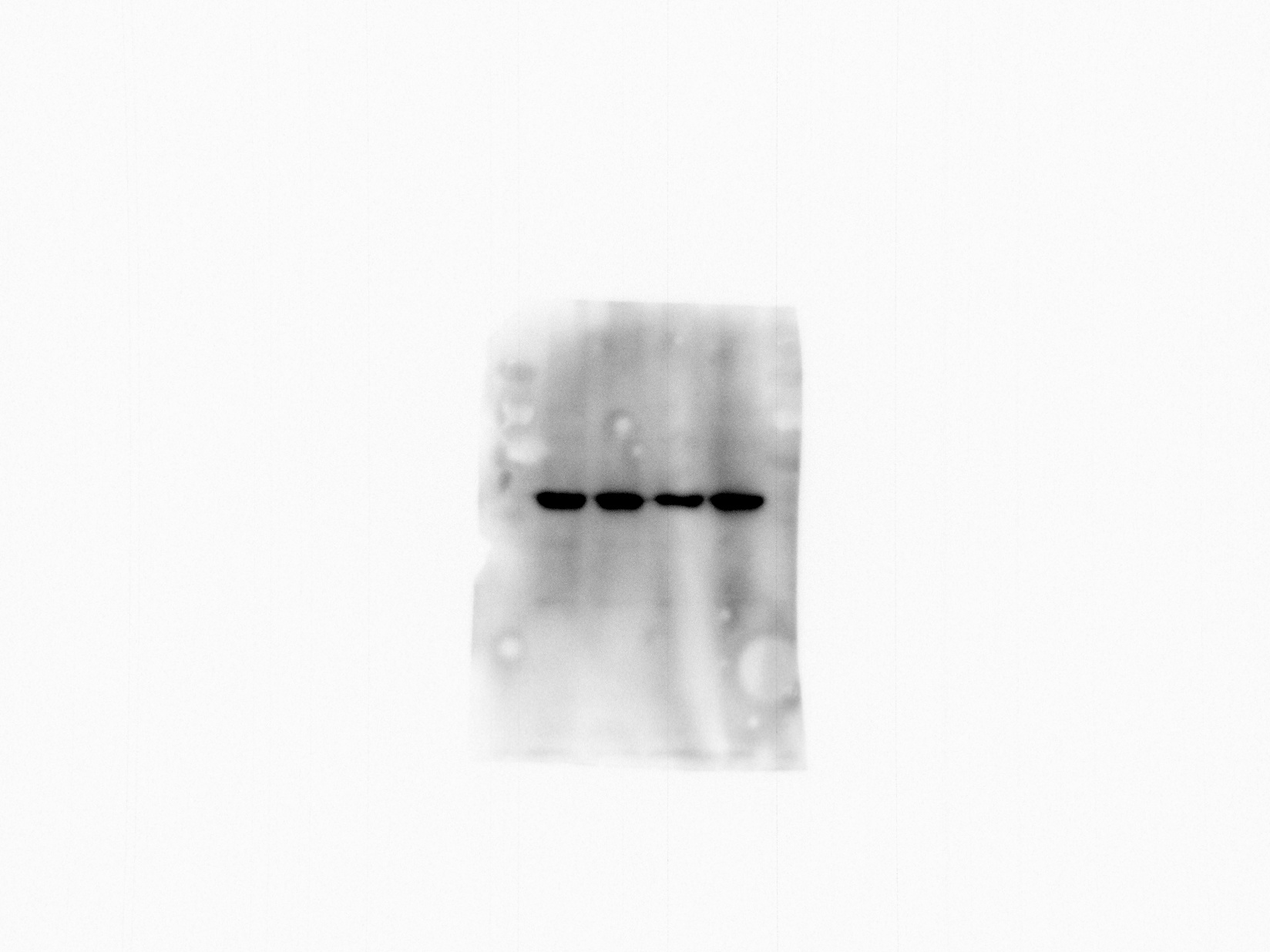

Supplement: Supplementary file 1 [file cancers-14-02406-s001.zip › Figure S7 original blots/Fig3G-tak1-1.jpg]

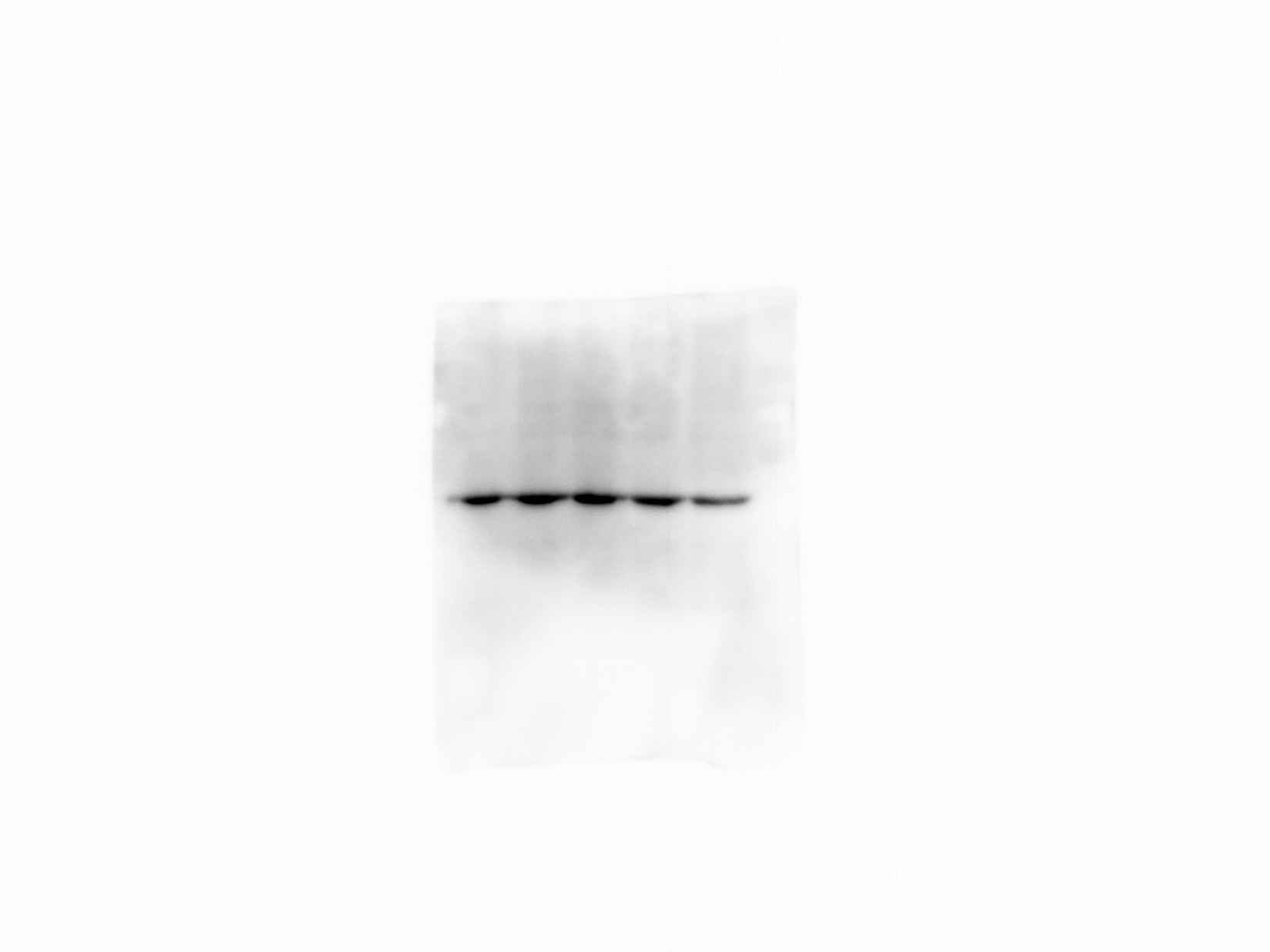

Supplement: Supplementary file 1 [file cancers-14-02406-s001.zip › Figure S7 original blots/Fig3G-TAK1-2.jpg]

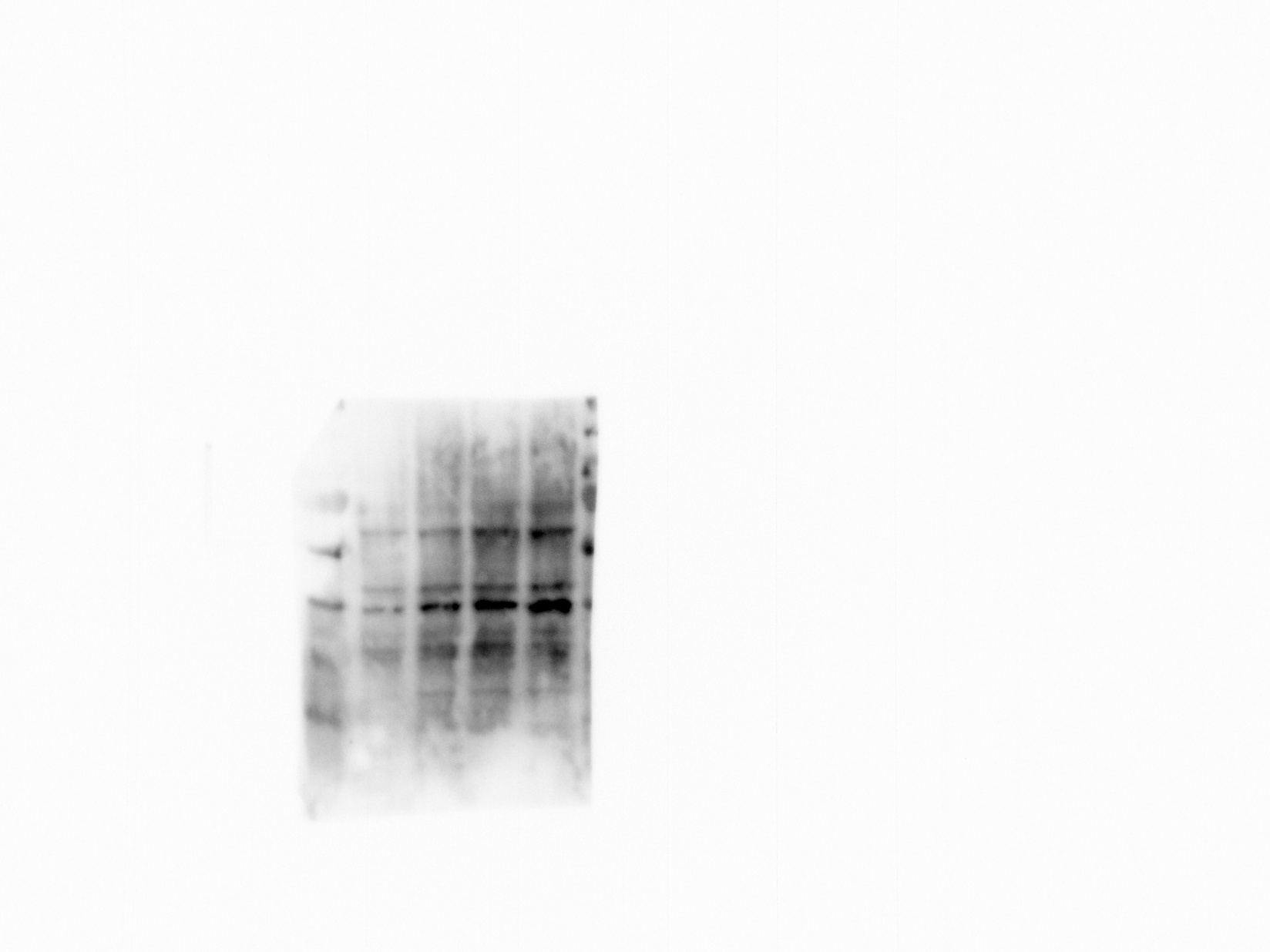

Supplement: Supplementary file 1 [file cancers-14-02406-s001.zip › Figure S7 original blots/Fig3H-AP1-1.jpg]

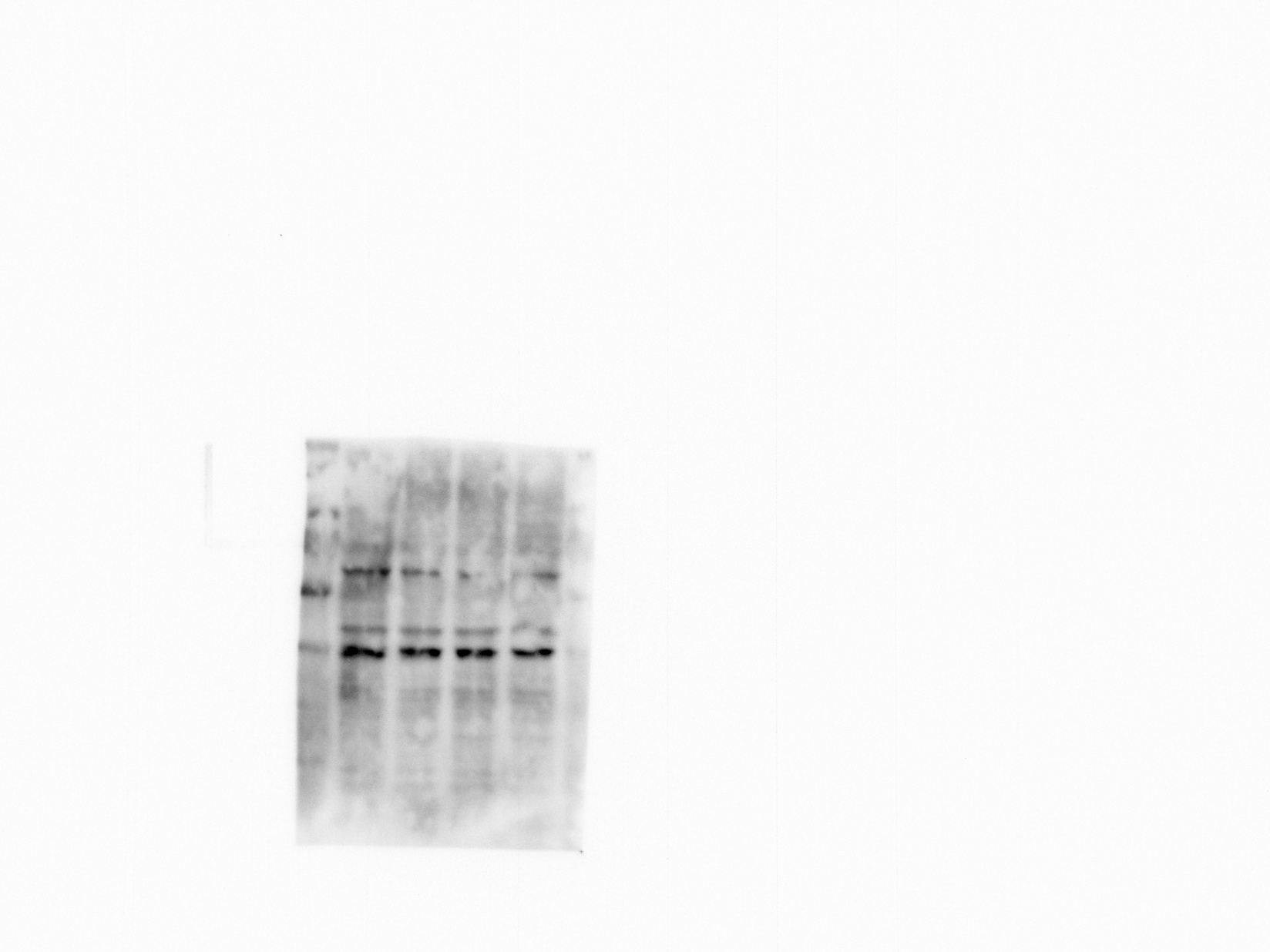

Supplement: Supplementary file 1 [file cancers-14-02406-s001.zip › Figure S7 original blots/Fig3H-AP1-2.jpg]

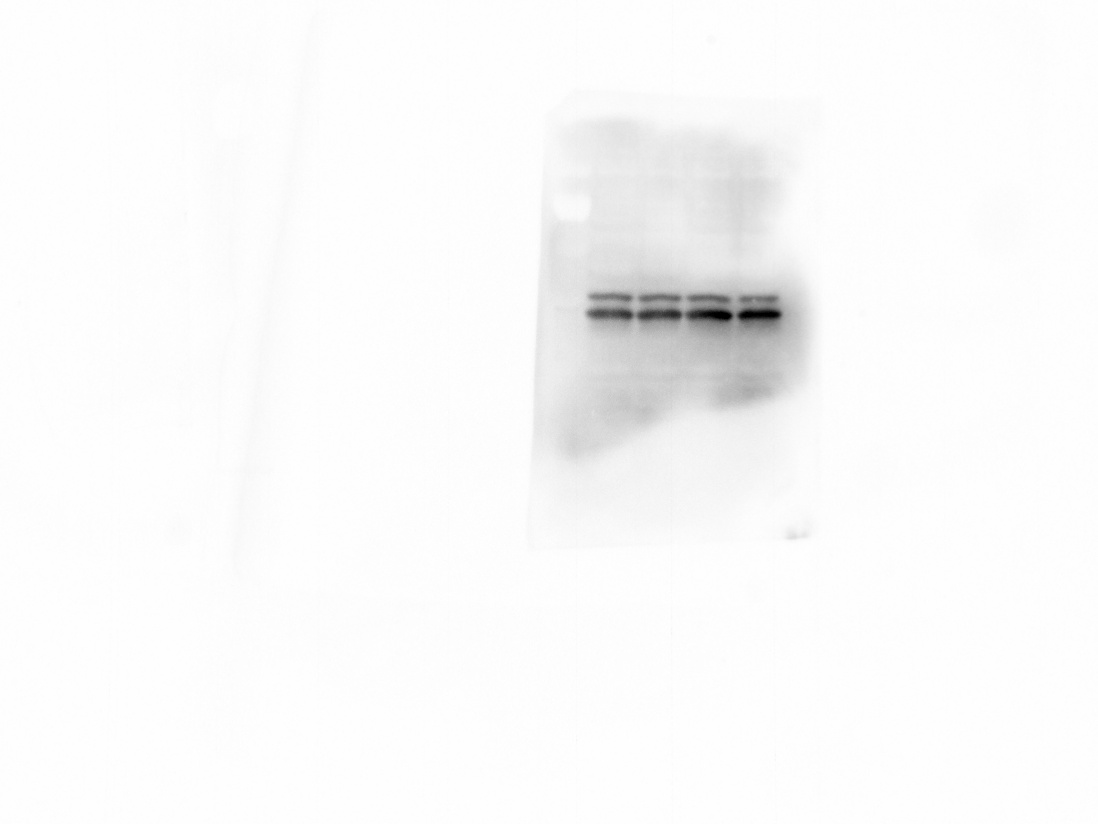

Supplement: Supplementary file 1 [file cancers-14-02406-s001.zip › Figure S7 original blots/Fig3H-ERK-1.jpg]

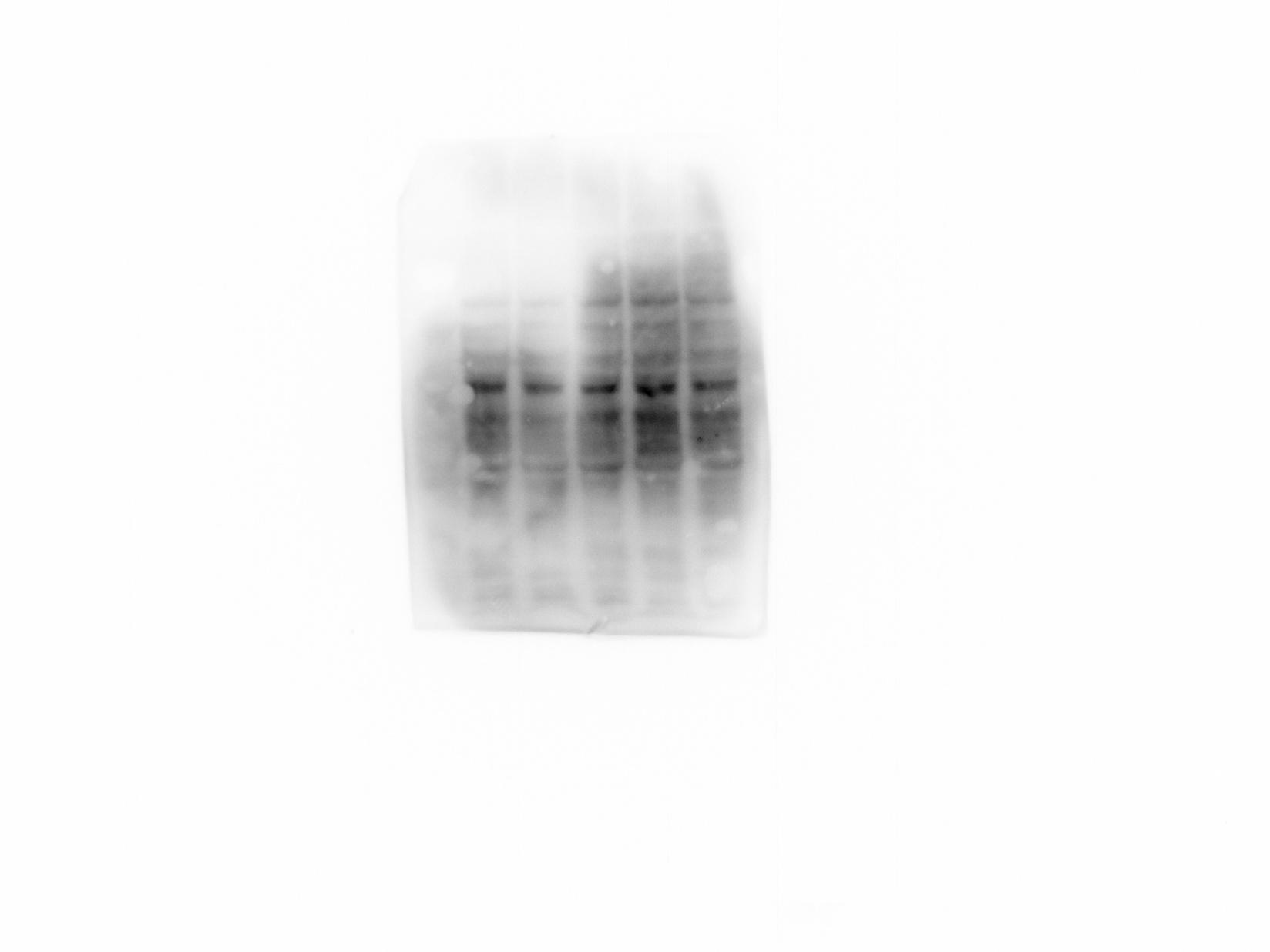

Supplement: Supplementary file 1 [file cancers-14-02406-s001.zip › Figure S7 original blots/Fig3H-erk-2.jpg]

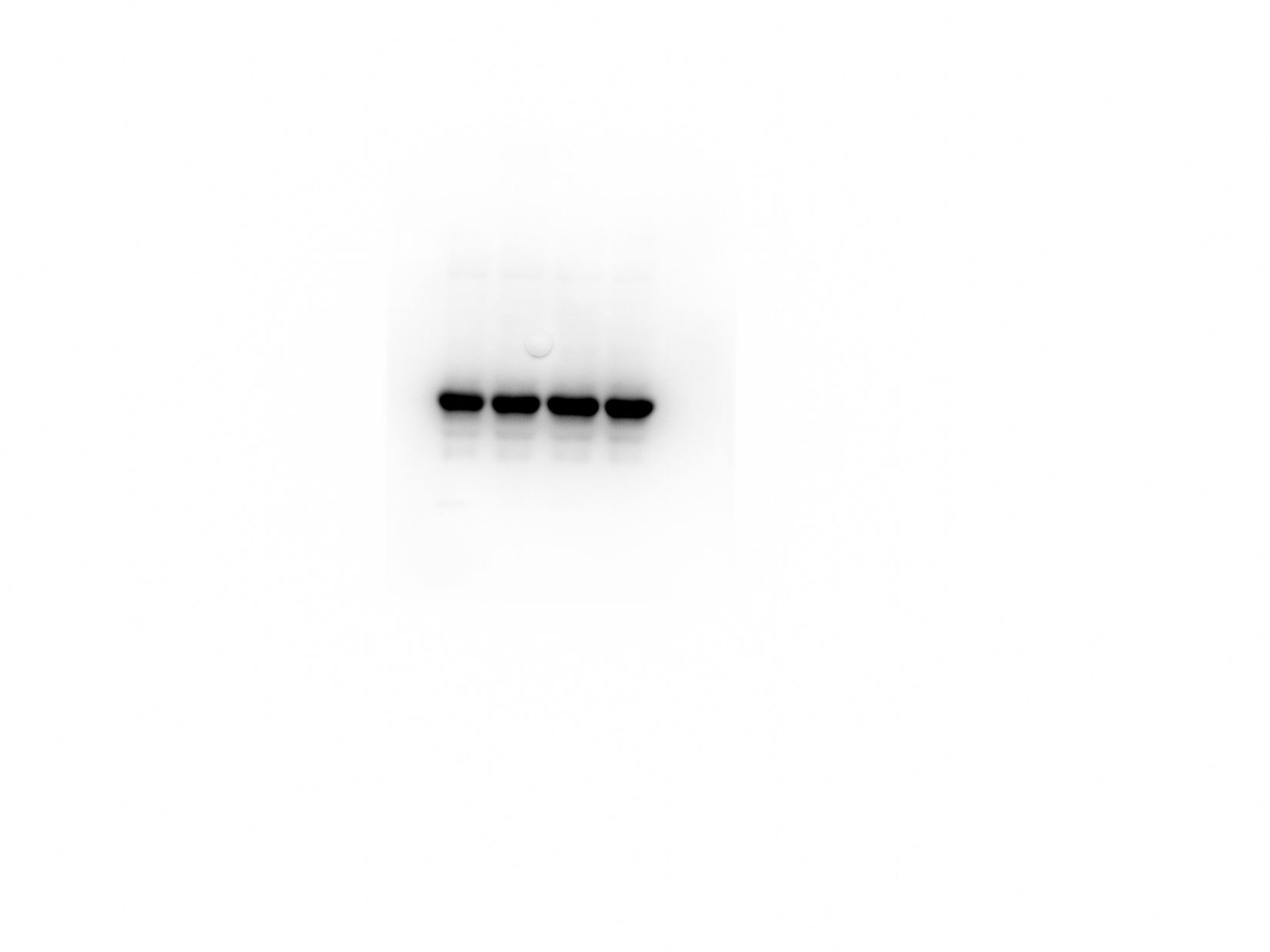

Supplement: Supplementary file 1 [file cancers-14-02406-s001.zip › Figure S7 original blots/Fig3H-gapdh-1.jpg]

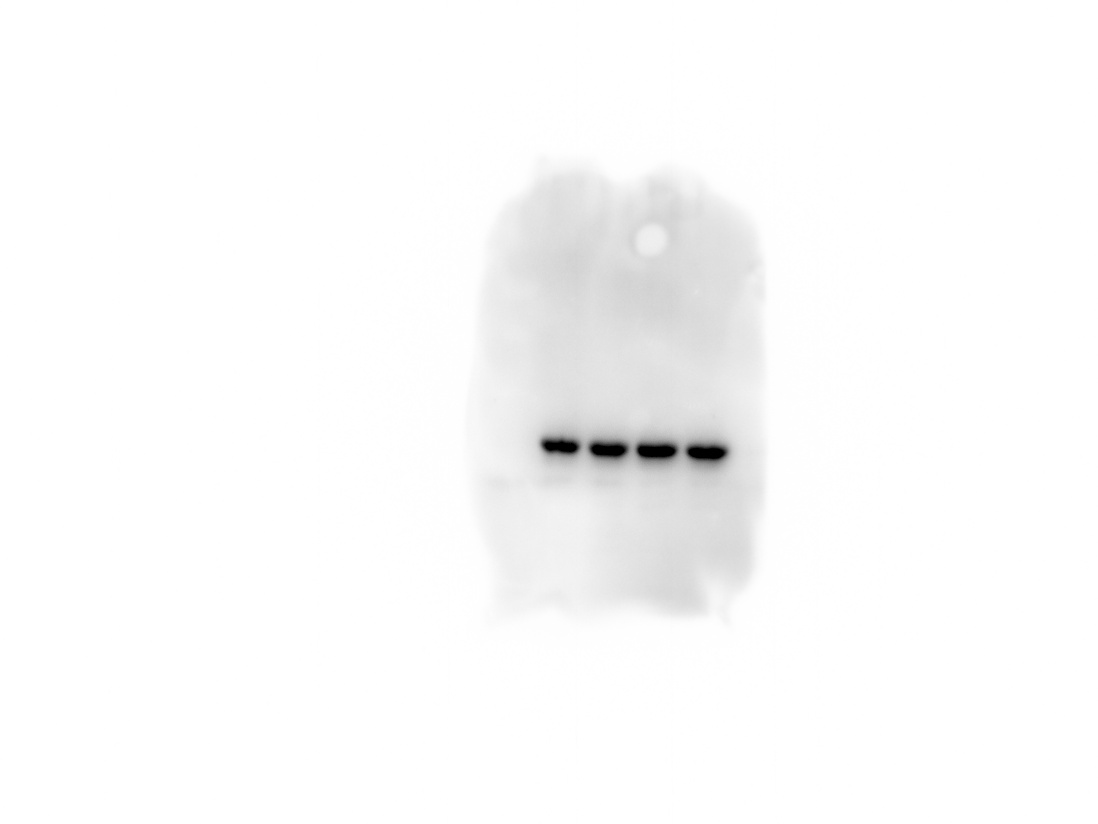

Supplement: Supplementary file 1 [file cancers-14-02406-s001.zip › Figure S7 original blots/Fig3H-gapdh-2.jpg]

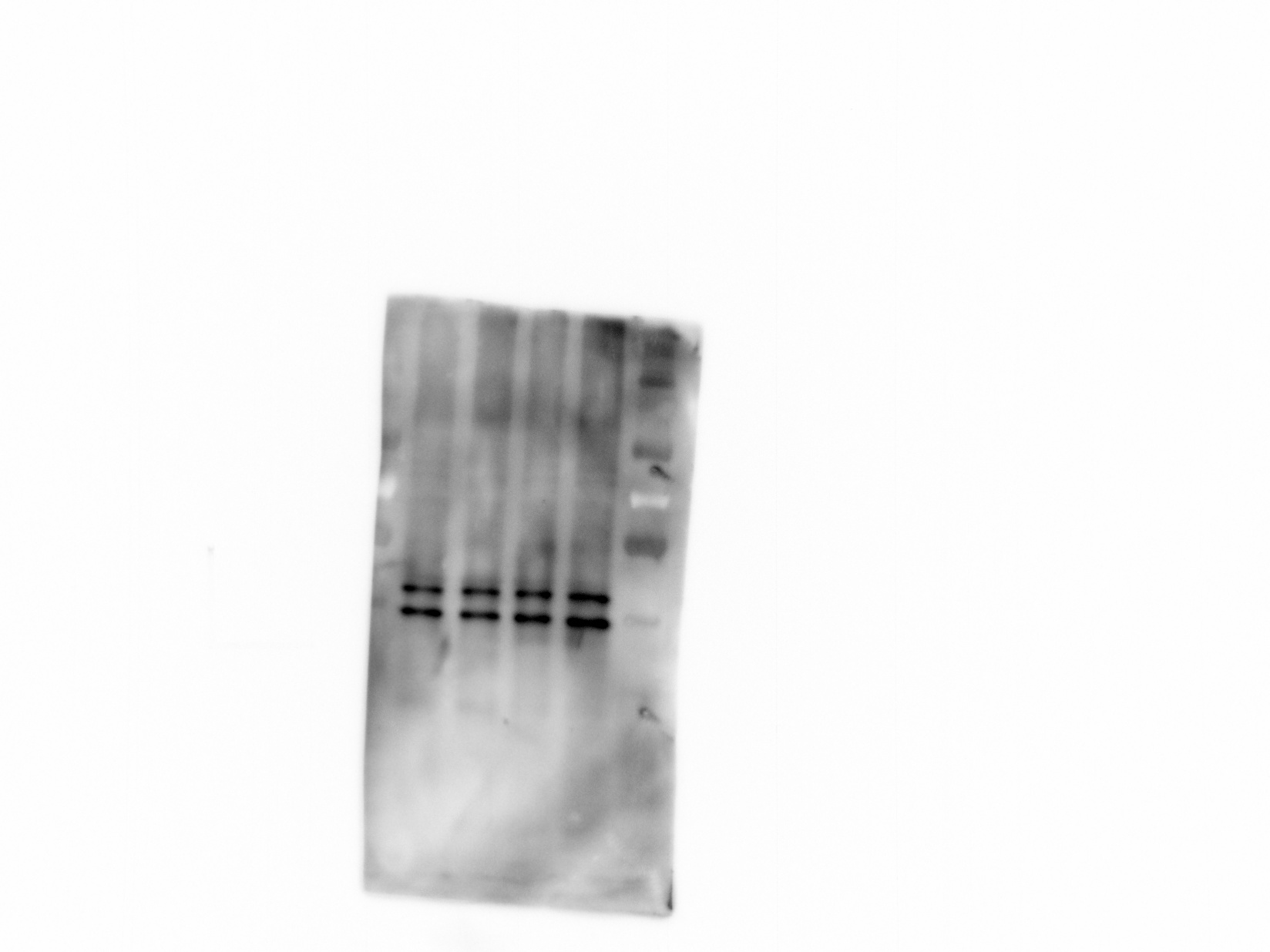

Supplement: Supplementary file 1 [file cancers-14-02406-s001.zip › Figure S7 original blots/Fig3H-JNK-1.jpg]

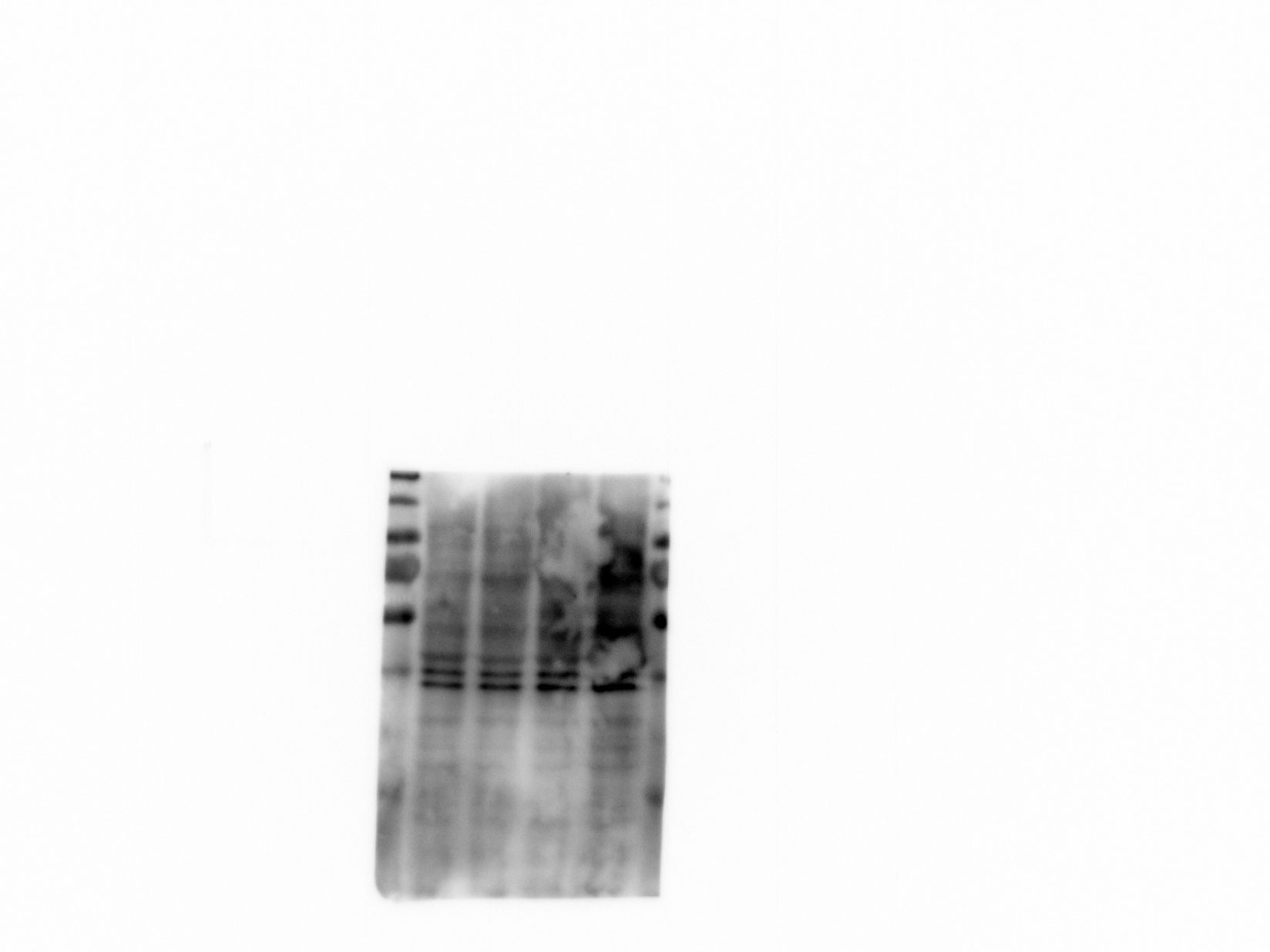

Supplement: Supplementary file 1 [file cancers-14-02406-s001.zip › Figure S7 original blots/Fig3H-JNK-2.jpg]

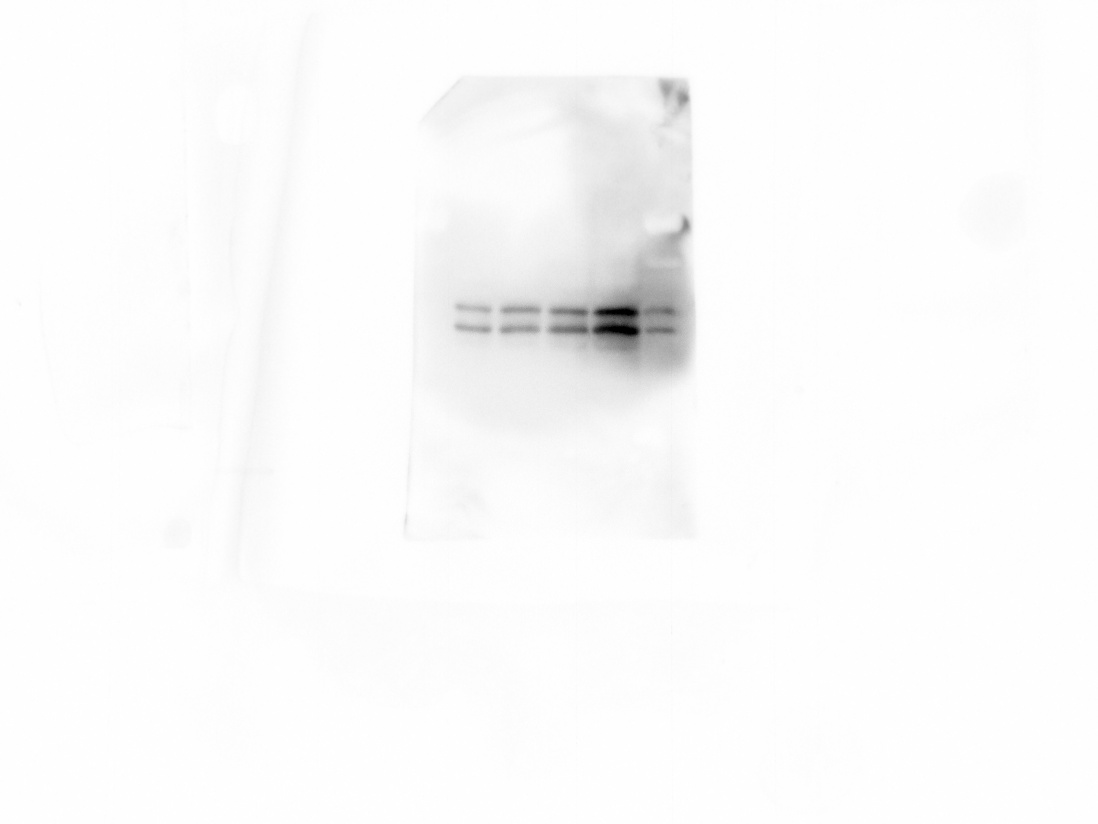

Supplement: Supplementary file 1 [file cancers-14-02406-s001.zip › Figure S7 original blots/Fig3H-perk-1.jpg]

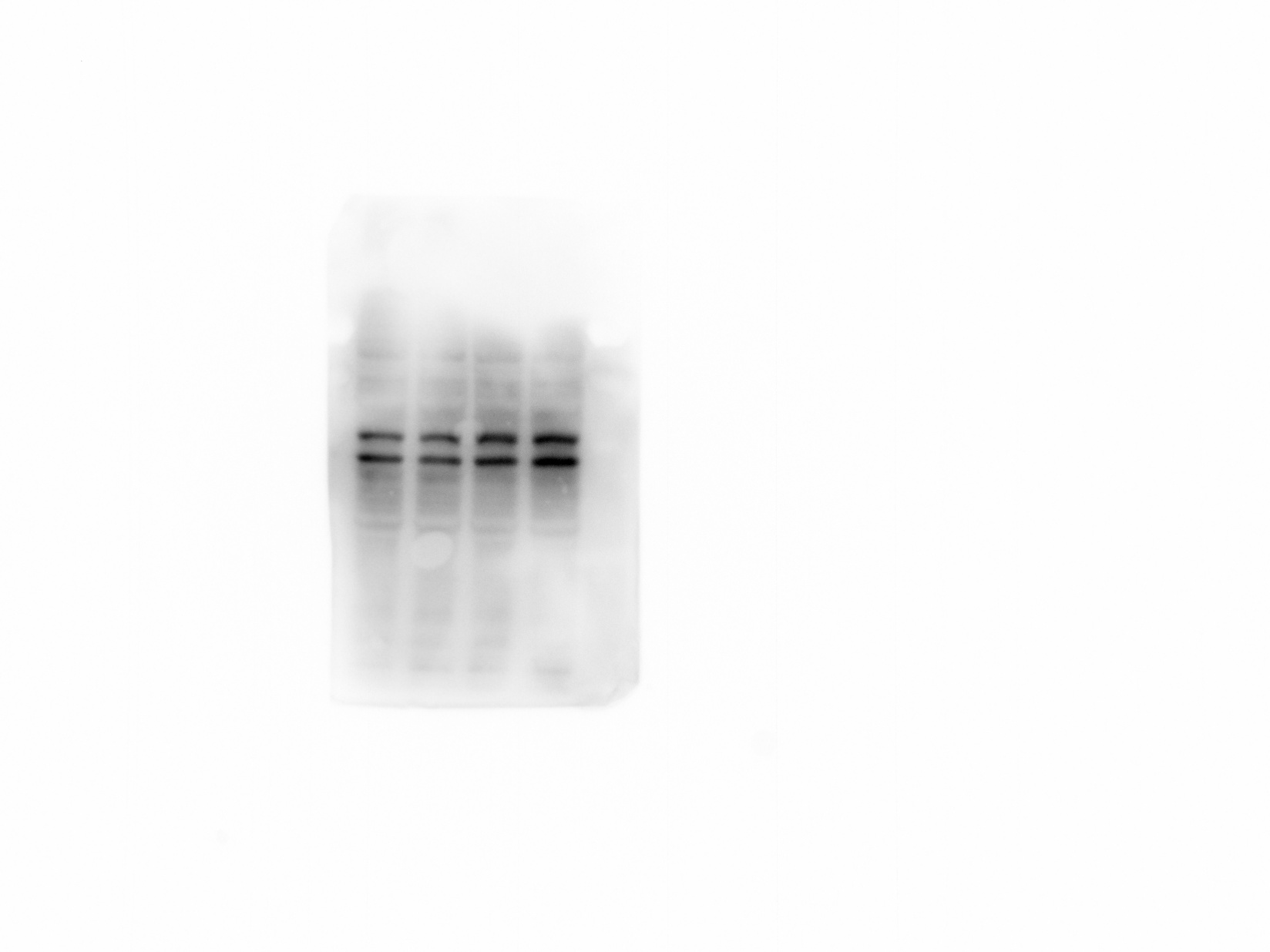

Supplement: Supplementary file 1 [file cancers-14-02406-s001.zip › Figure S7 original blots/Fig3H-perk-2.jpg]

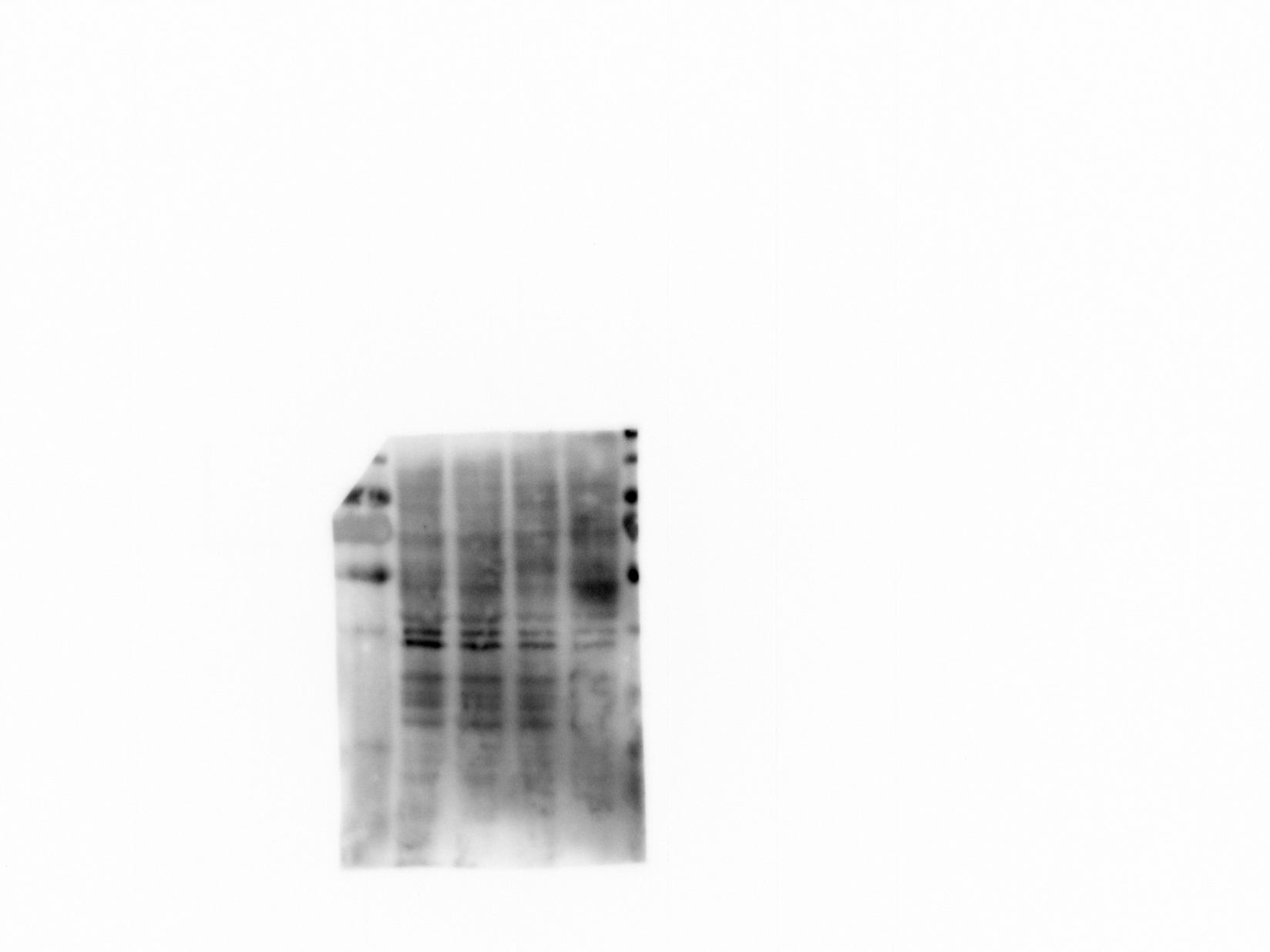

Supplement: Supplementary file 1 [file cancers-14-02406-s001.zip › Figure S7 original blots/Fig3H-PJNK-1.jpg]

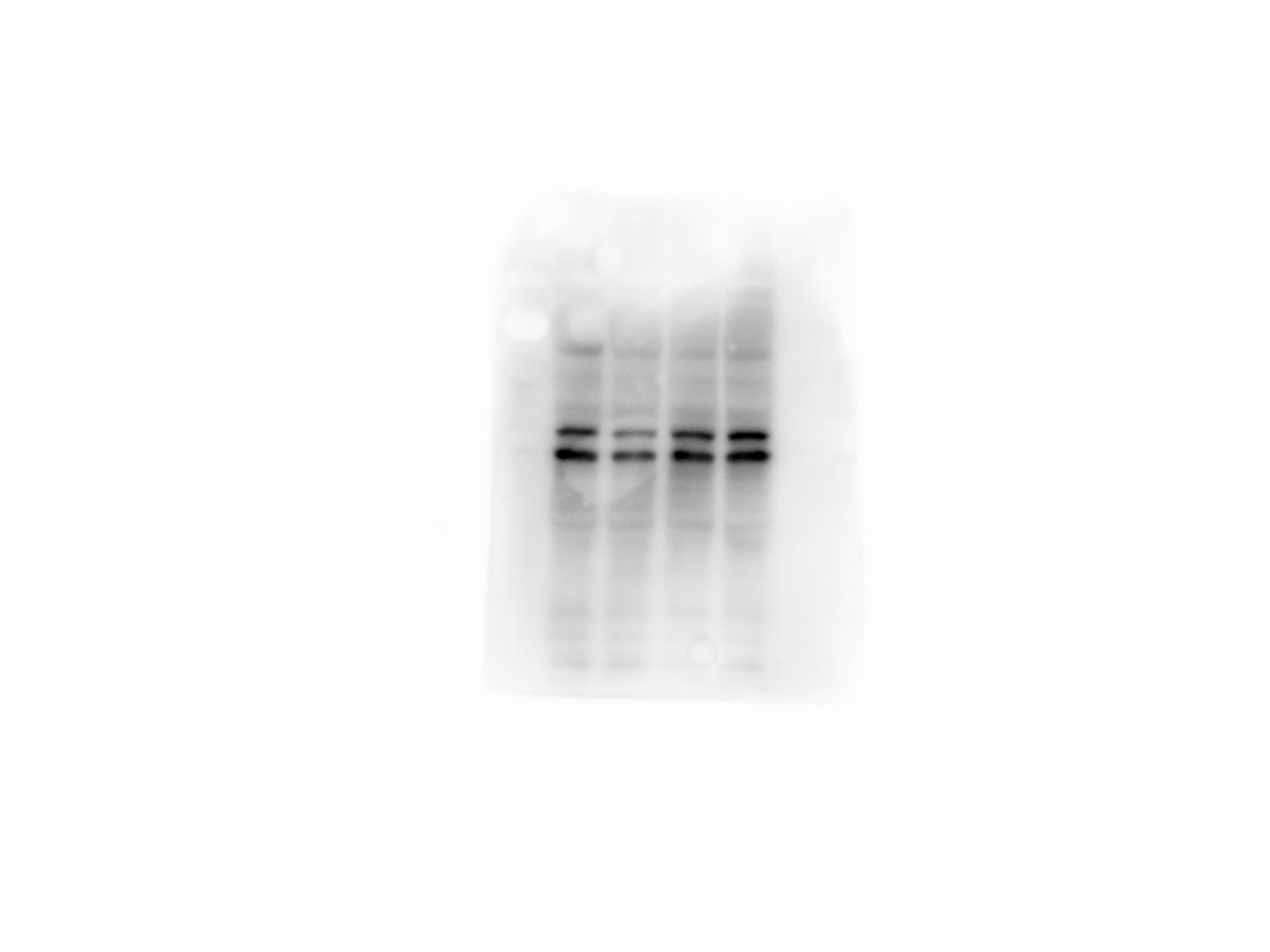

Supplement: Supplementary file 1 [file cancers-14-02406-s001.zip › Figure S7 original blots/Fig3H-pJNK-2.jpg]

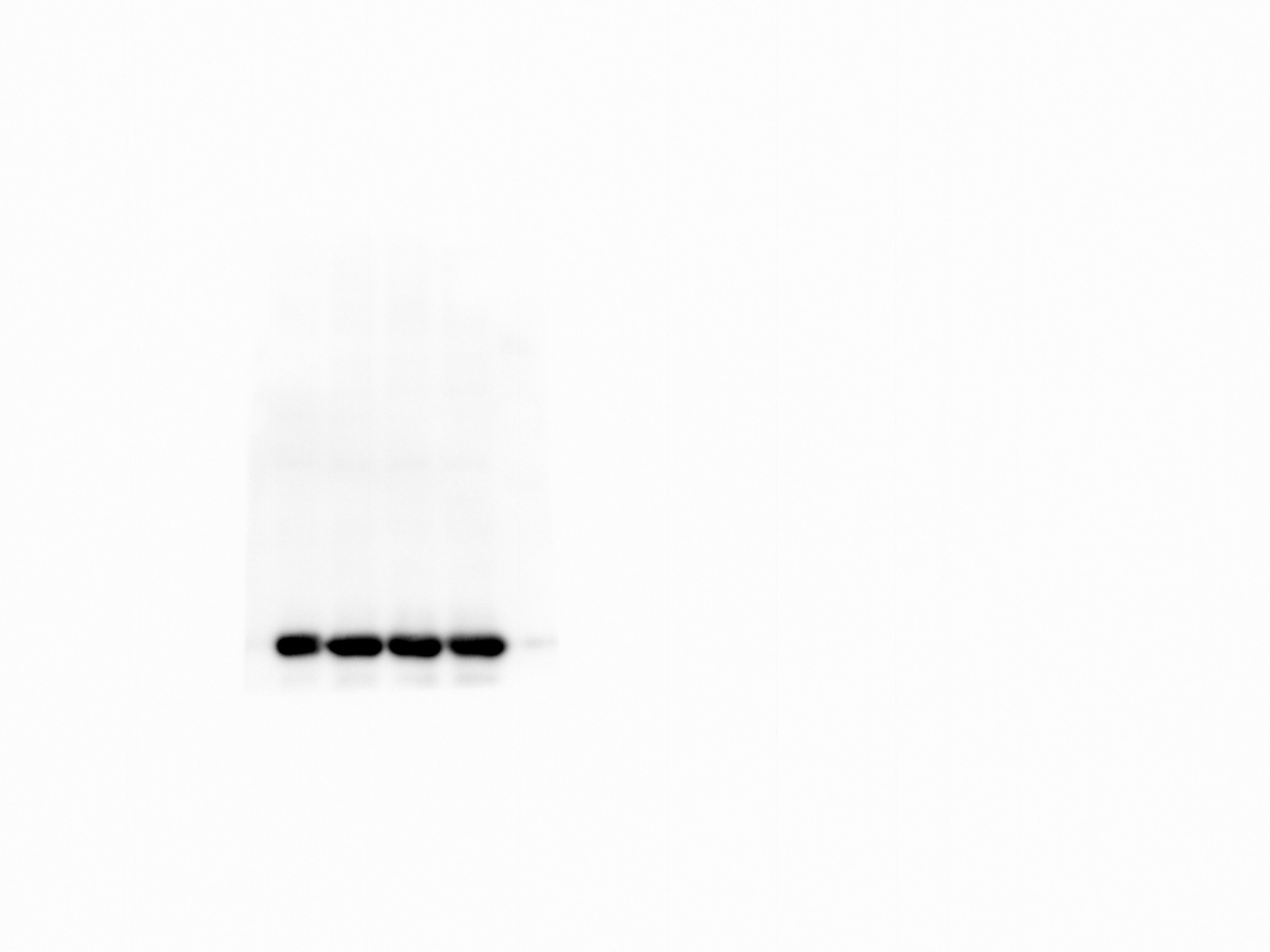

Supplement: Supplementary file 1 [file cancers-14-02406-s001.zip › Figure S7 original blots/Fig4C-gapdh.jpg]

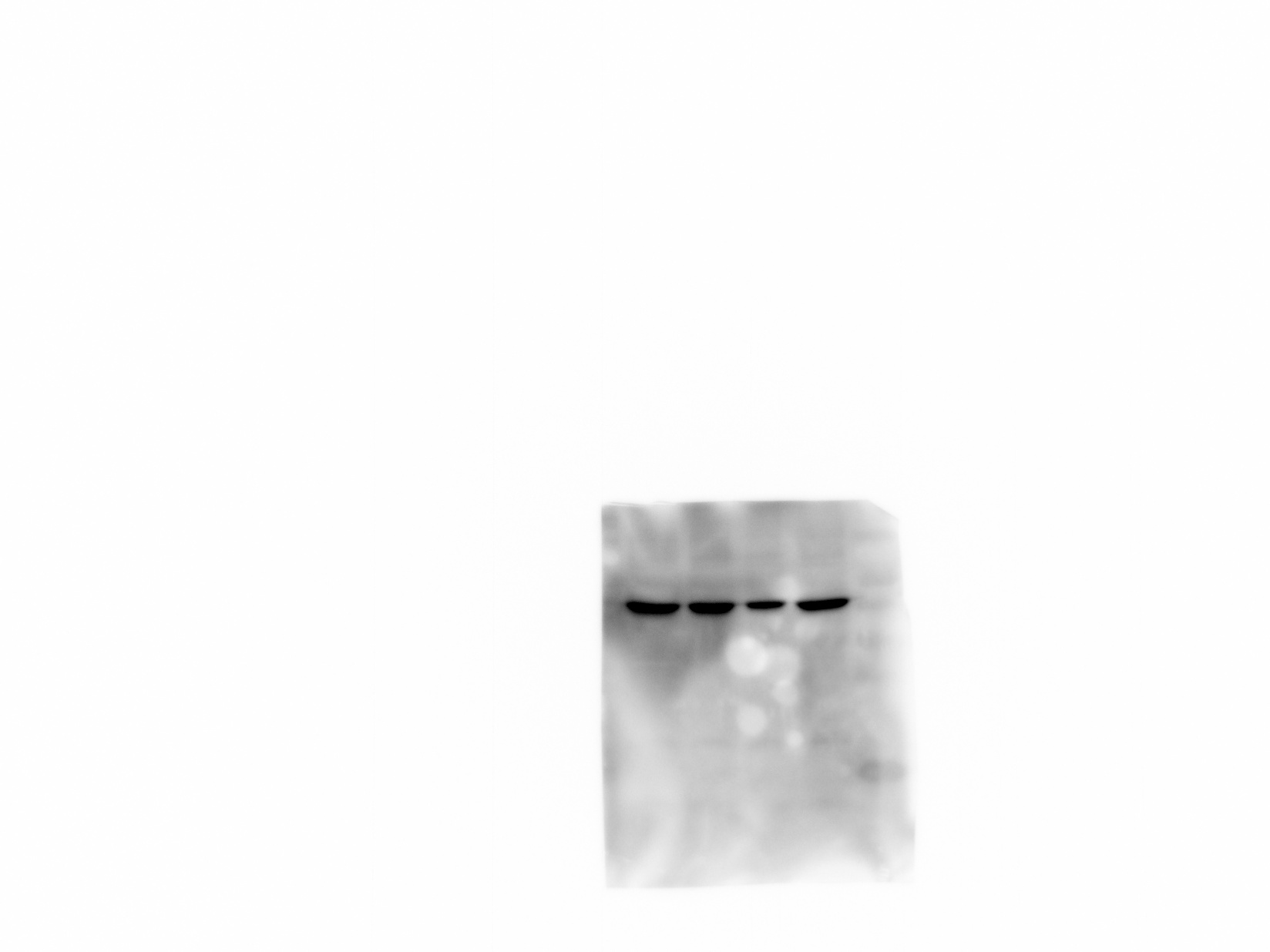

Supplement: Supplementary file 1 [file cancers-14-02406-s001.zip › Figure S7 original blots/Fig4C-SMA.jpg]

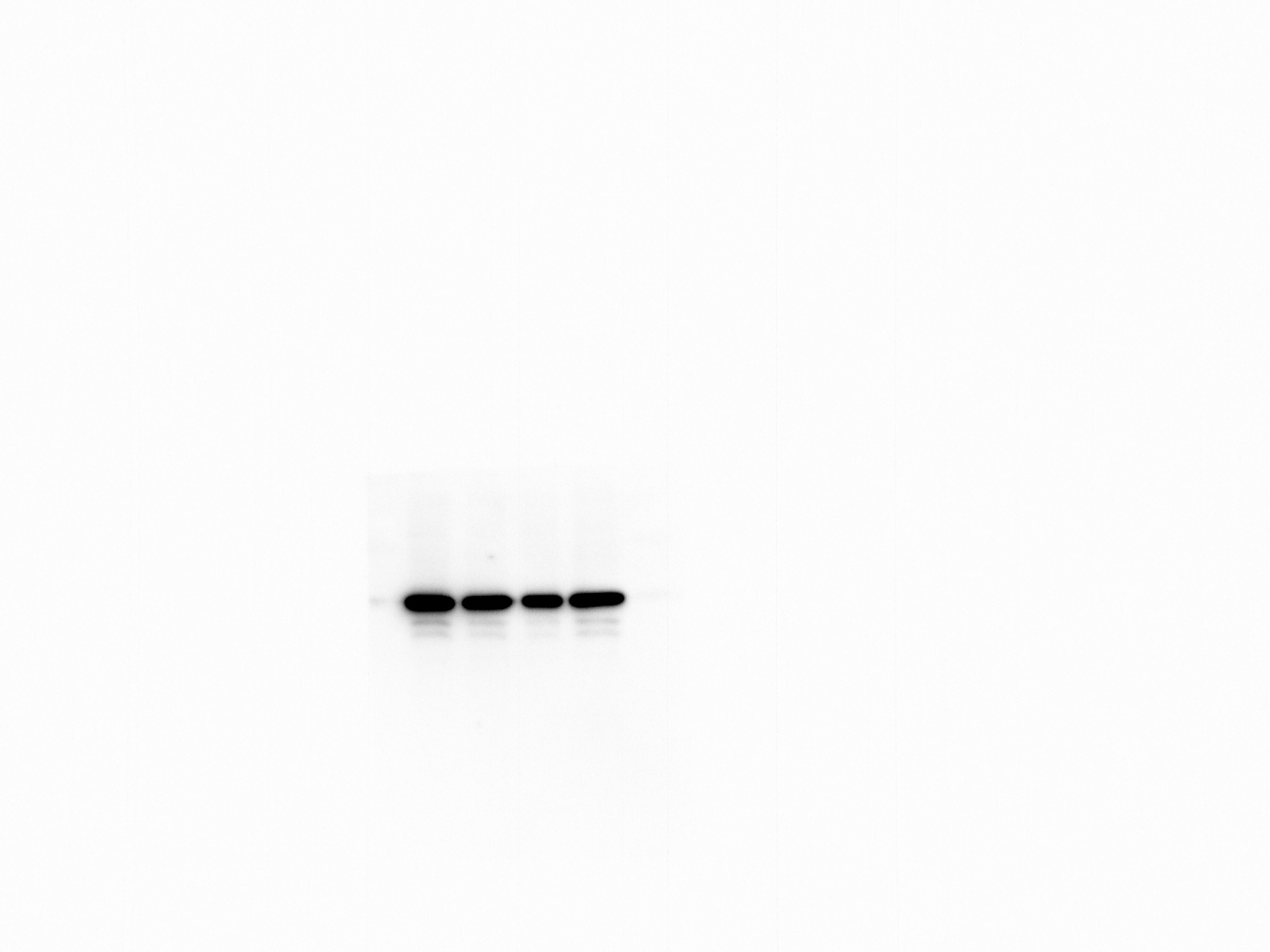

Supplement: Supplementary file 1 [file cancers-14-02406-s001.zip › Figure S7 original blots/Fig4C-vegfa.jpg]

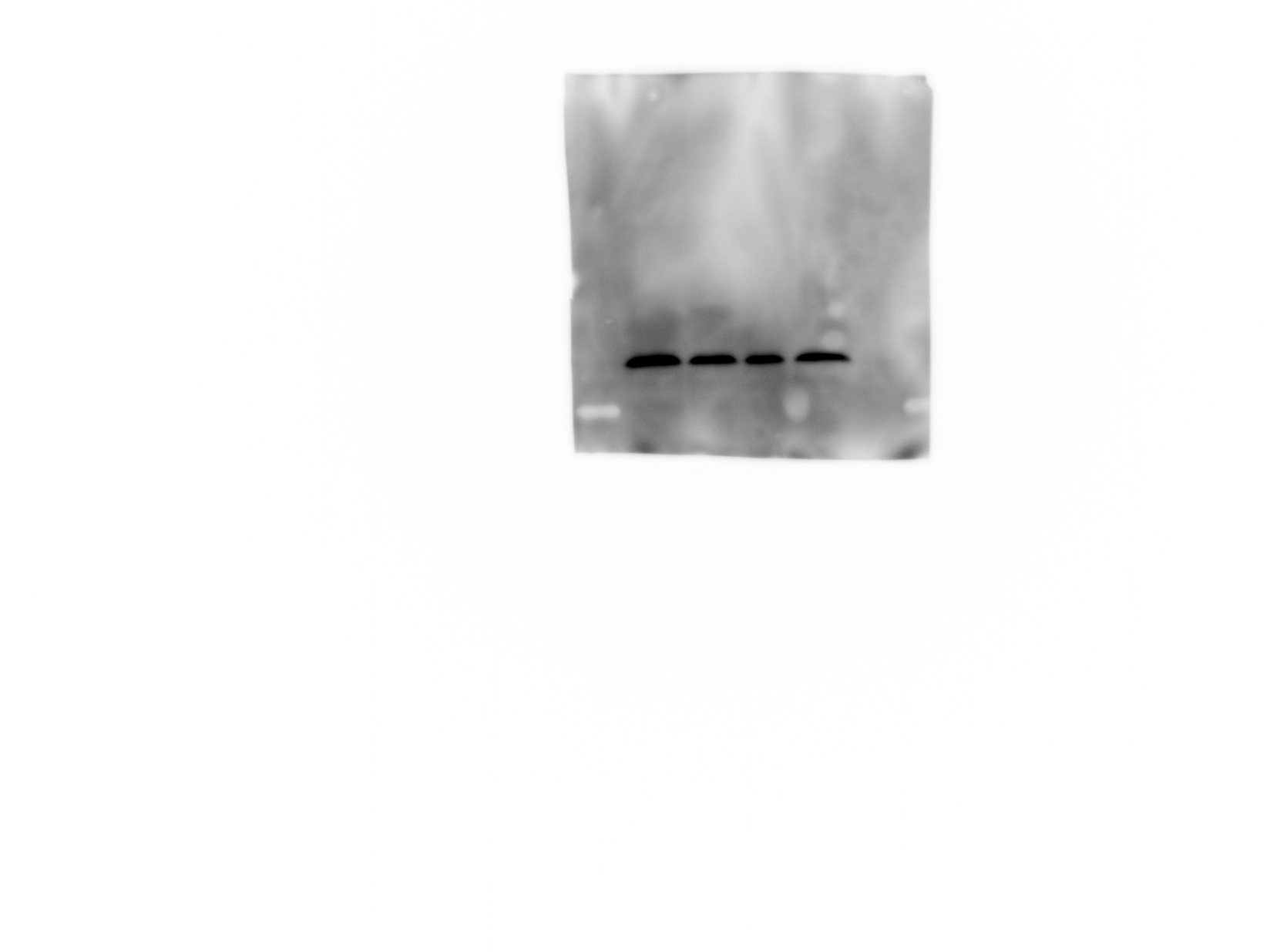

Supplement: Supplementary file 1 [file cancers-14-02406-s001.zip › Figure S7 original blots/Fig4Cs100a4.jpg]

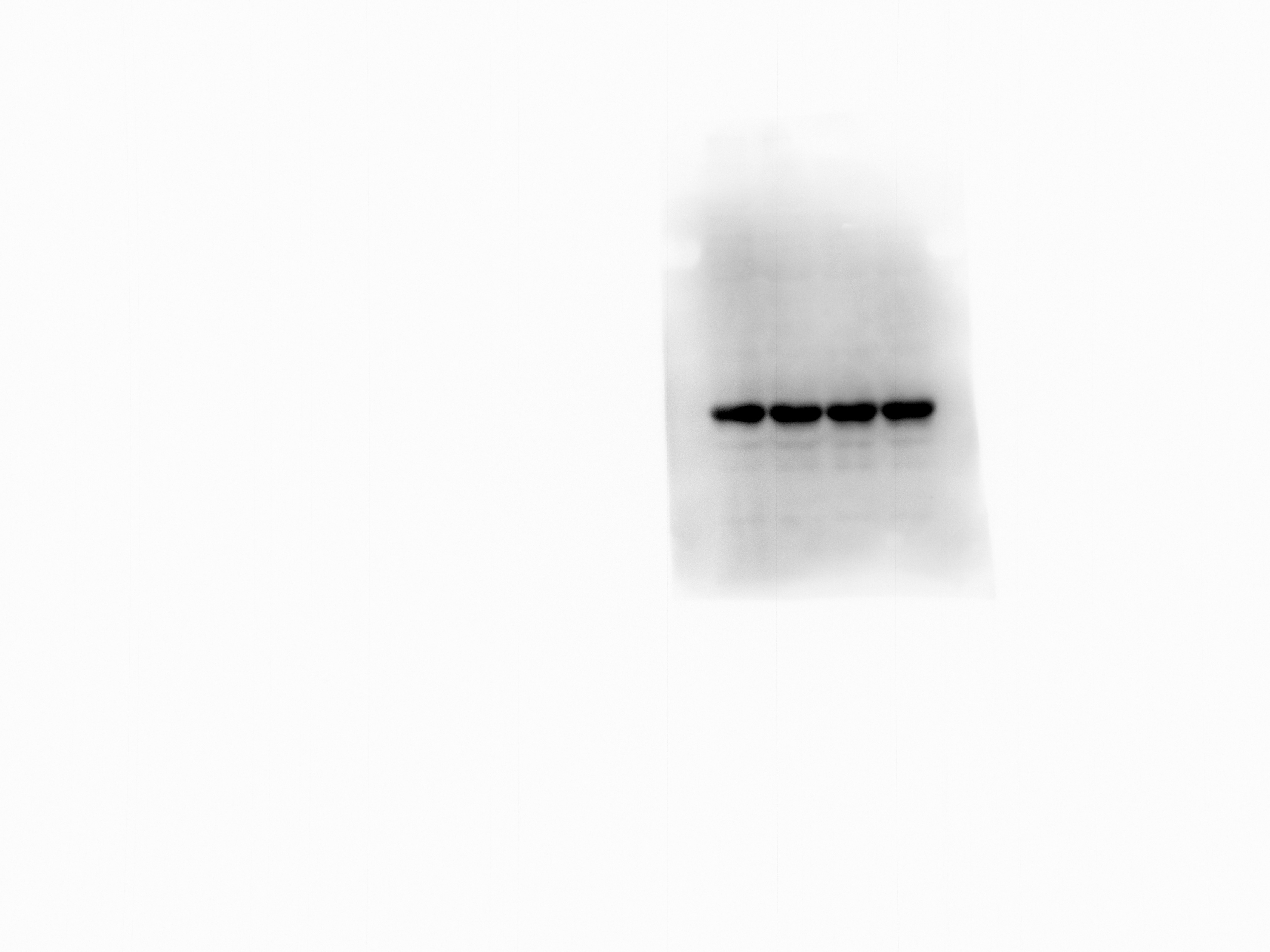

Supplement: Supplementary file 1 [file cancers-14-02406-s001.zip › Figure S7 original blots/Fig5E-gapdh.jpg]

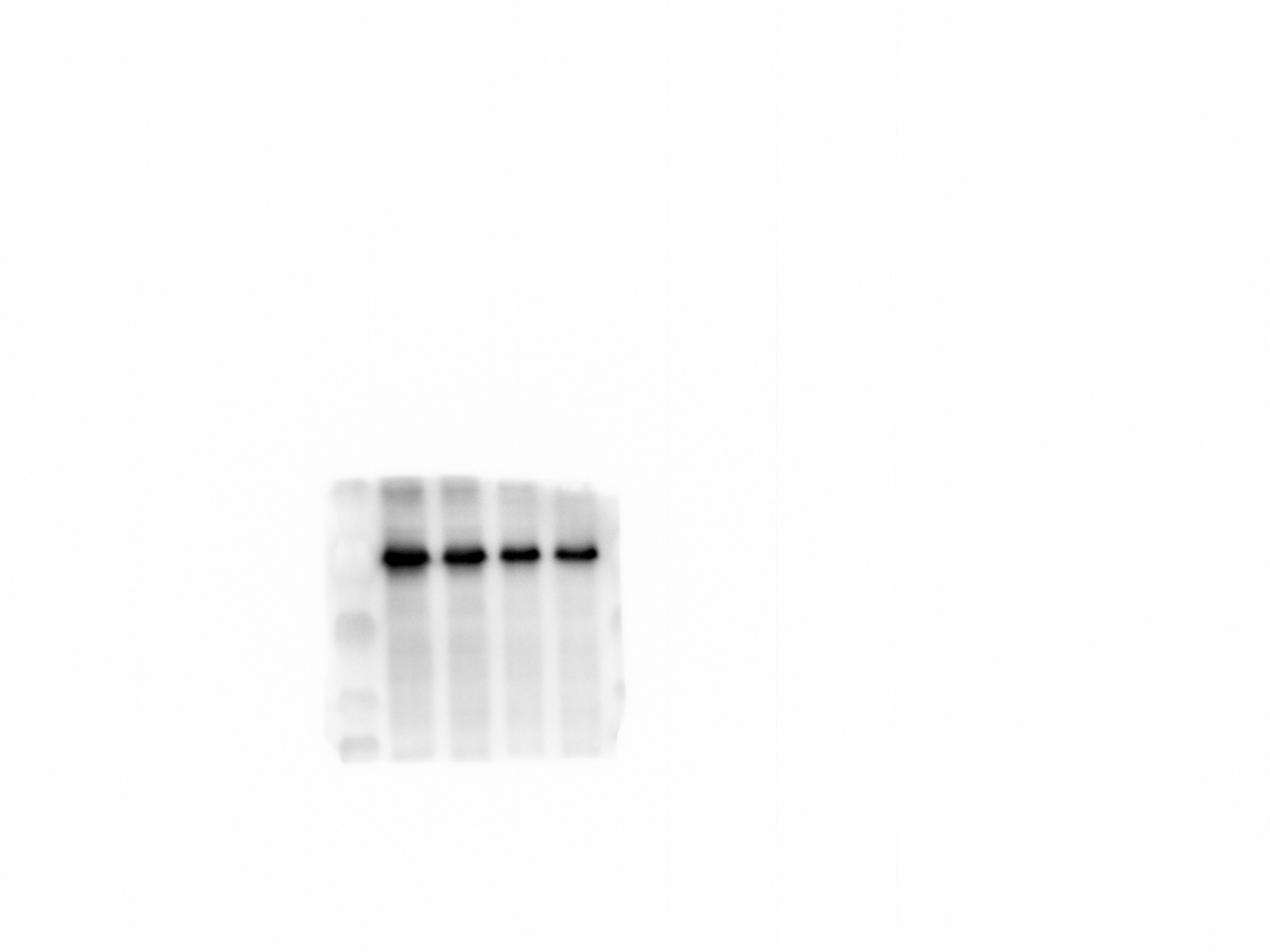

Supplement: Supplementary file 1 [file cancers-14-02406-s001.zip › Figure S7 original blots/Fig5E-hif-1.jpg]

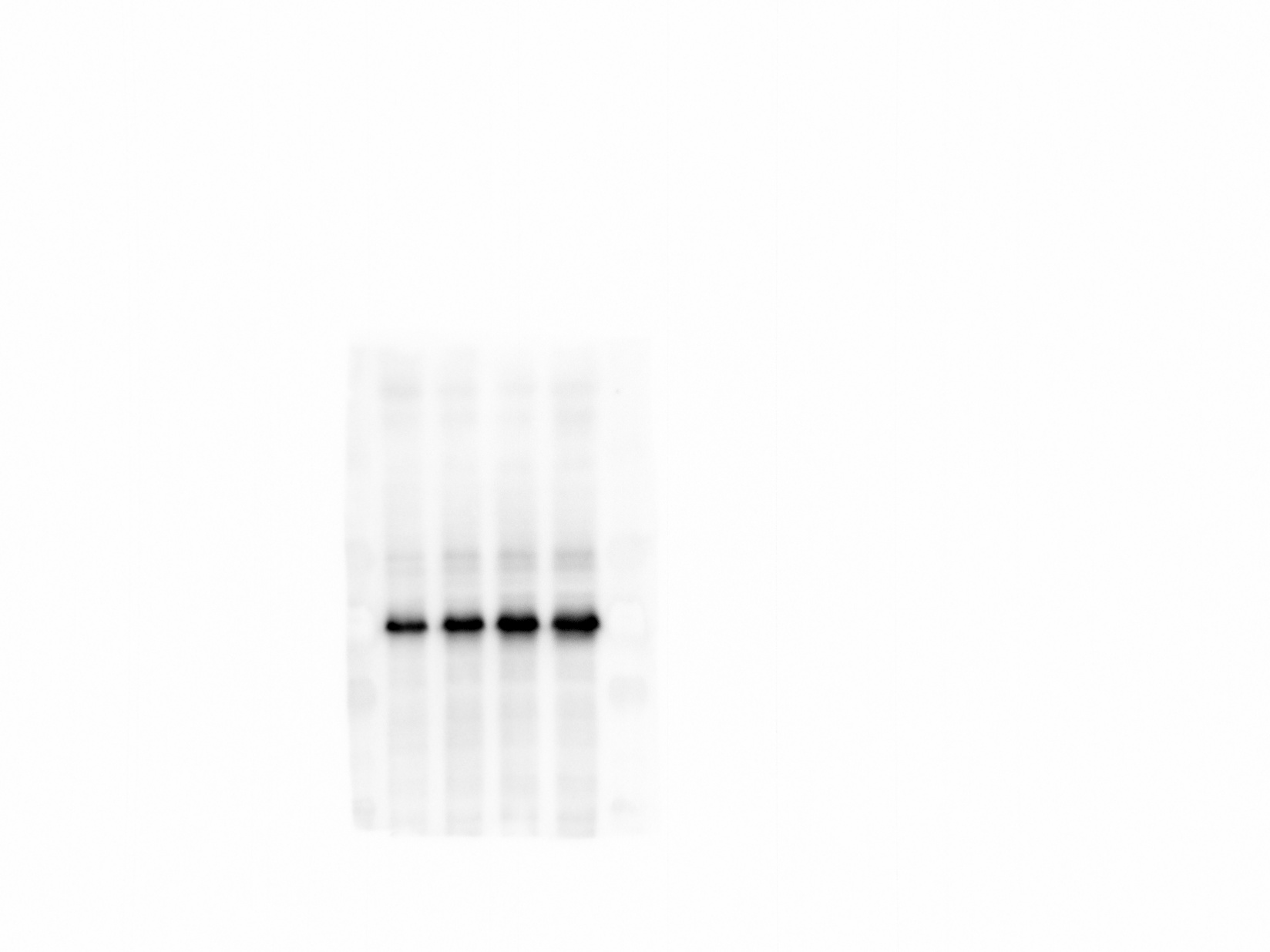

Supplement: Supplementary file 1 [file cancers-14-02406-s001.zip › Figure S7 original blots/Fig5E-jab1.jpg]

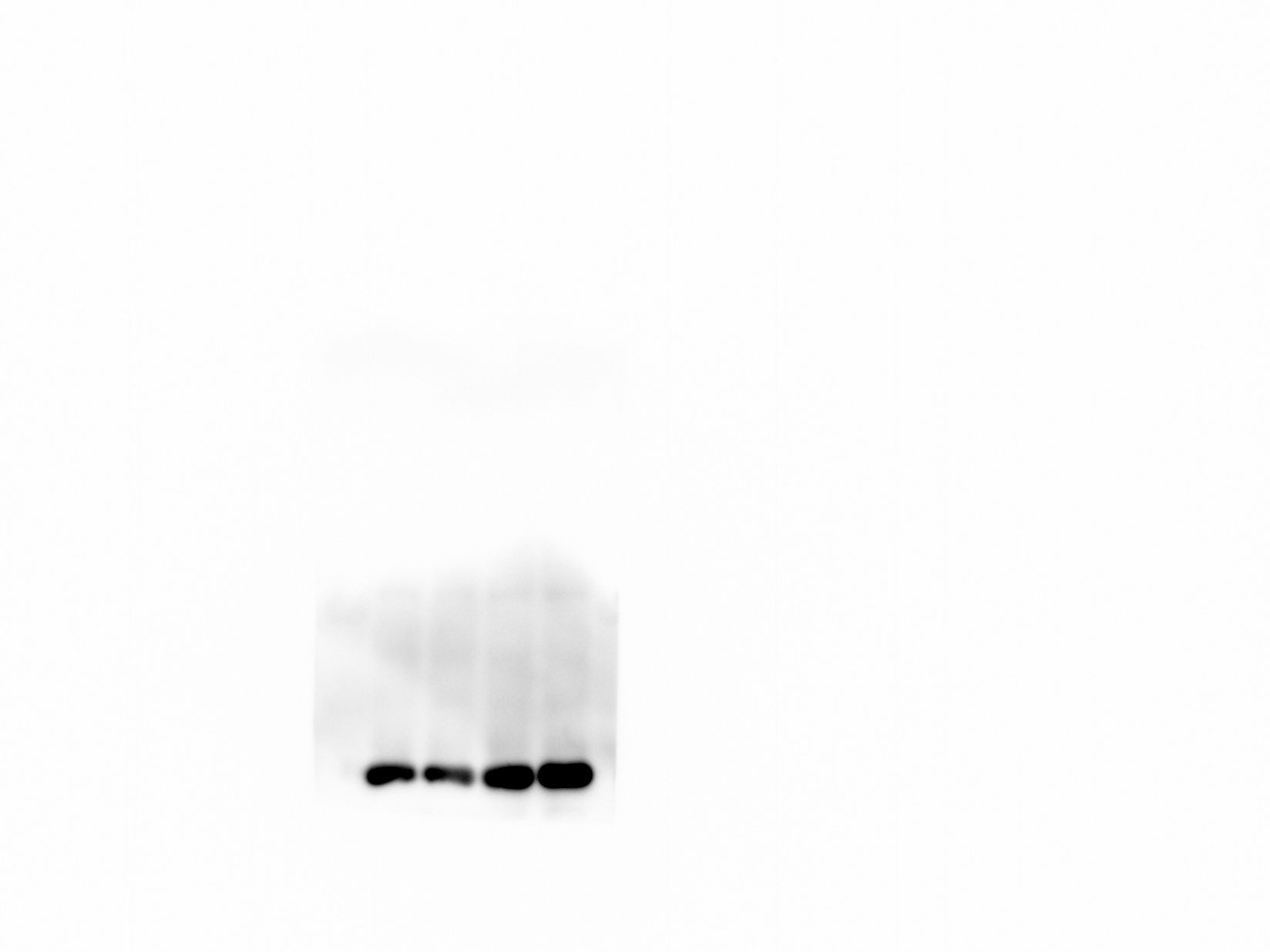

Supplement: Supplementary file 1 [file cancers-14-02406-s001.zip › Figure S7 original blots/Fig5E-pp53.jpg]

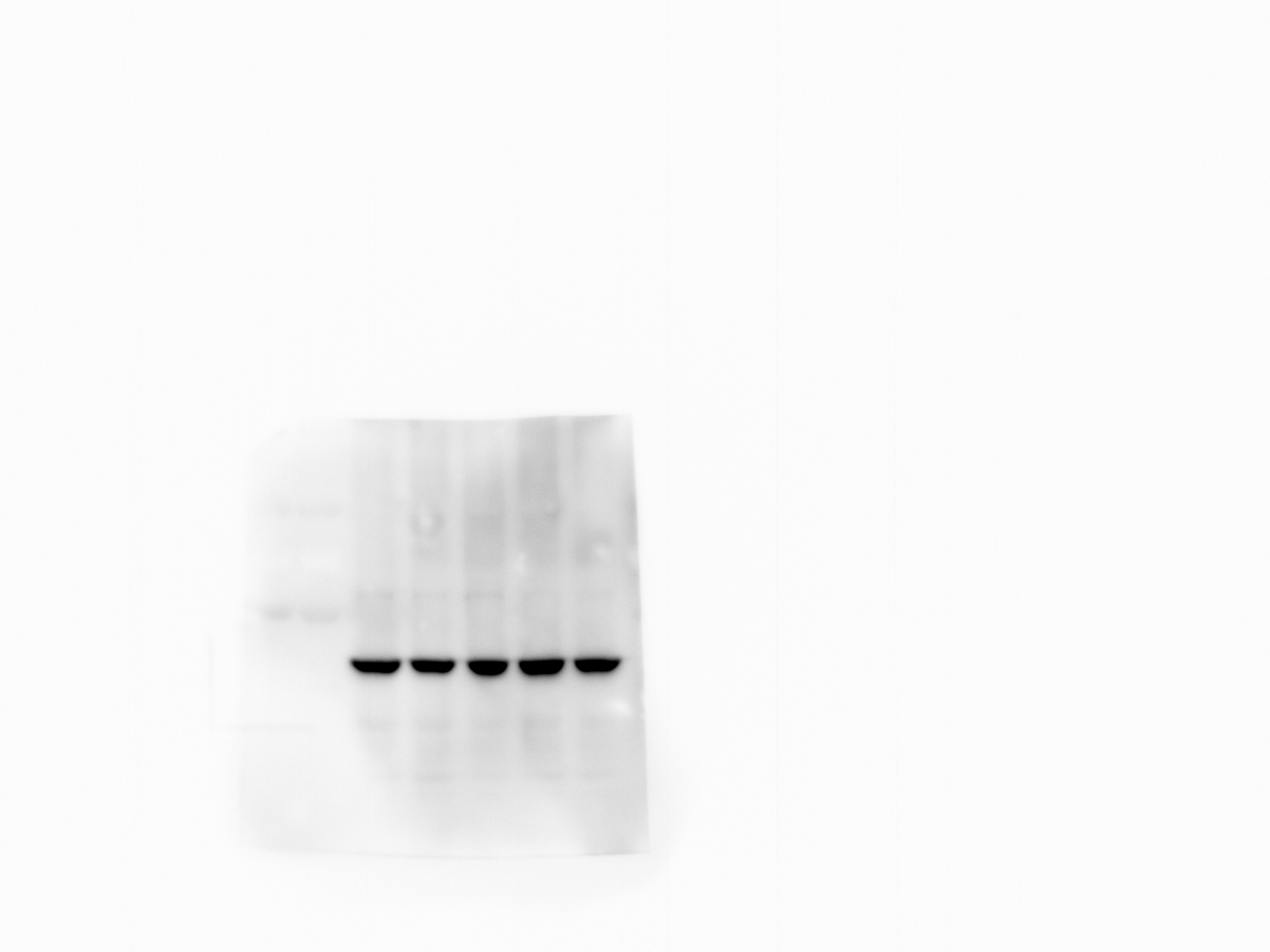

Supplement: Supplementary file 1 [file cancers-14-02406-s001.zip › Figure S7 original blots/Fig6C-akt.jpg]

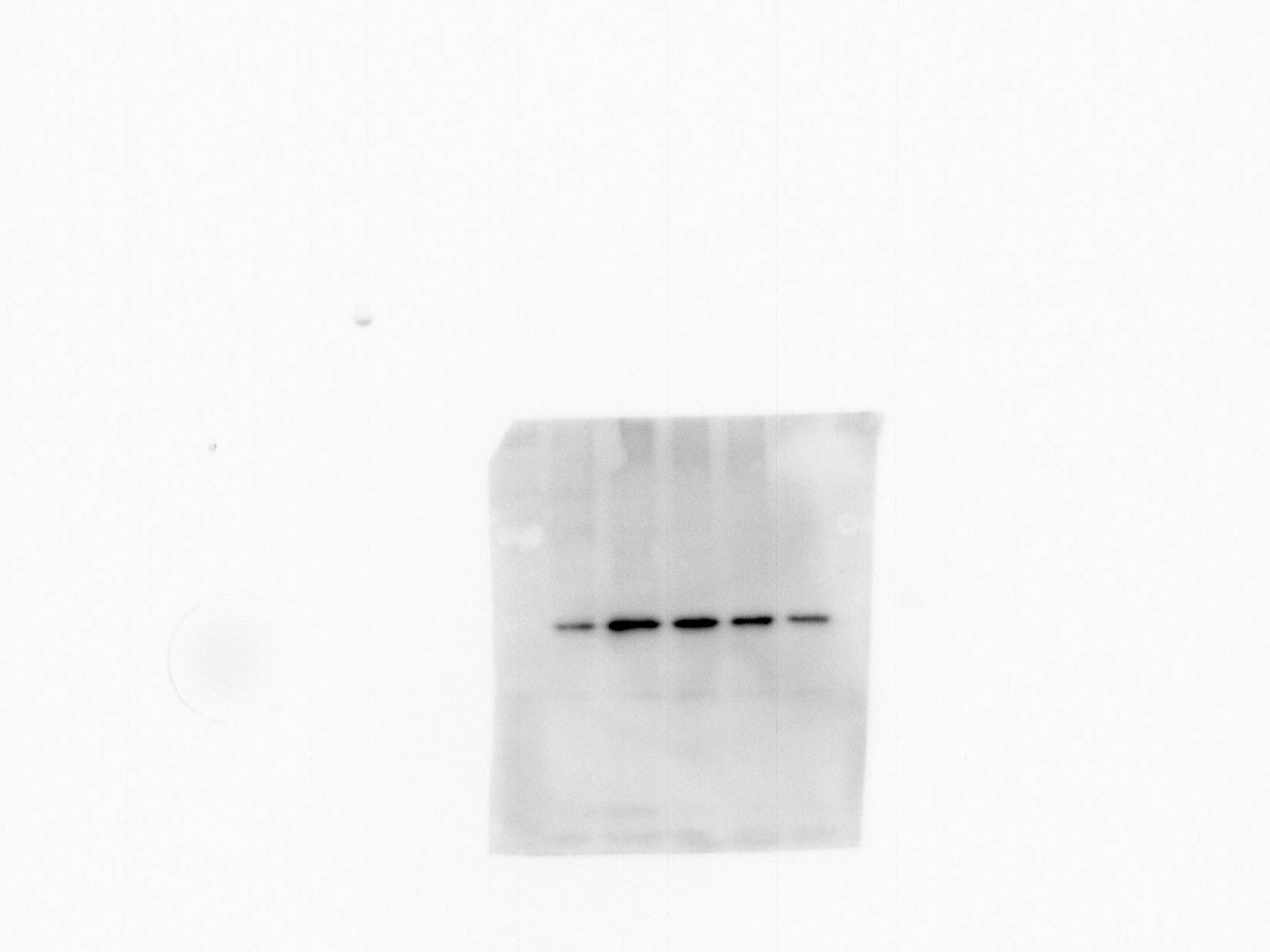

Supplement: Supplementary file 1 [file cancers-14-02406-s001.zip › Figure S7 original blots/Fig6C-AP1.jpg]

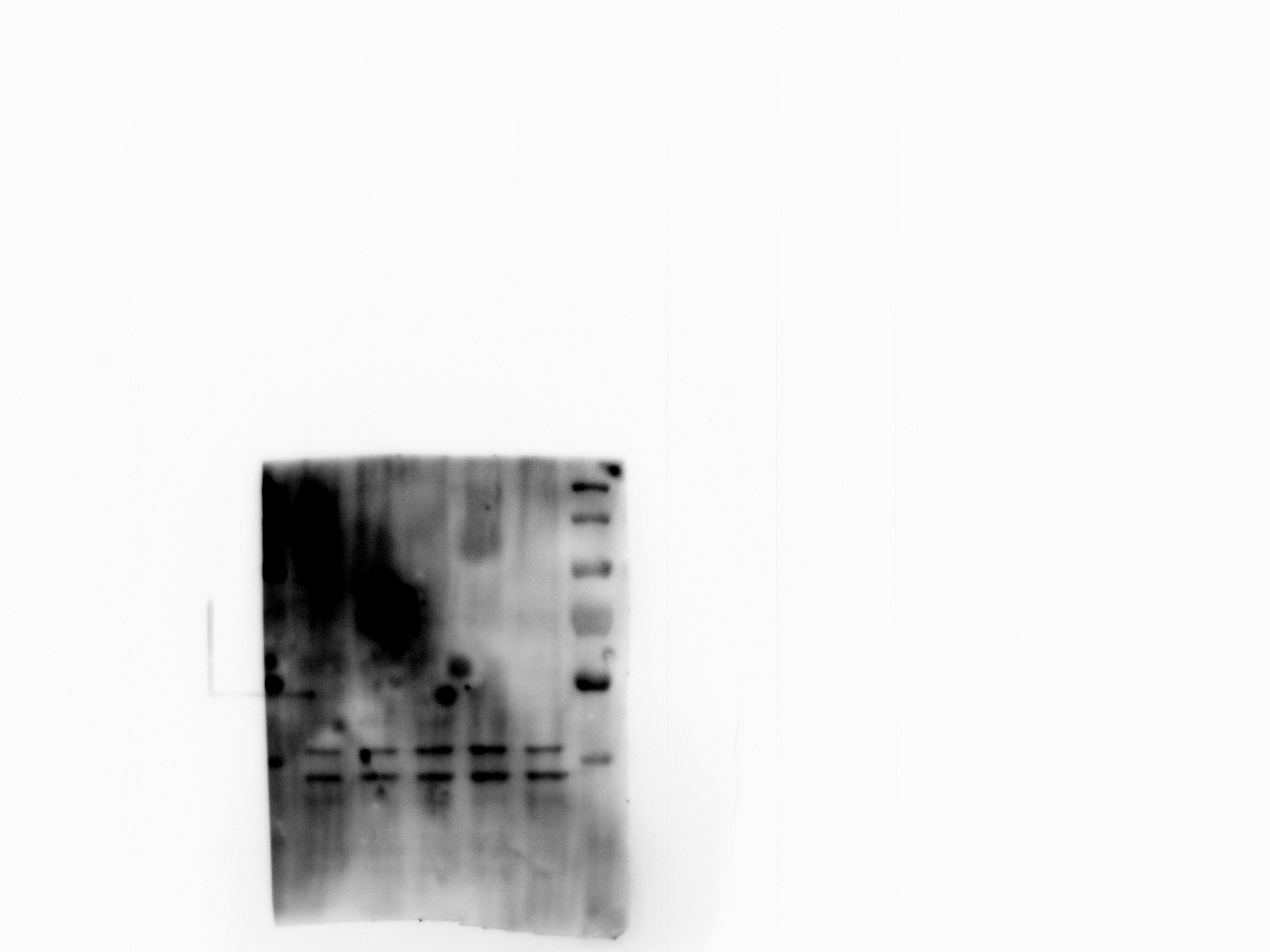

Supplement: Supplementary file 1 [file cancers-14-02406-s001.zip › Figure S7 original blots/Fig6C-erk.jpg]

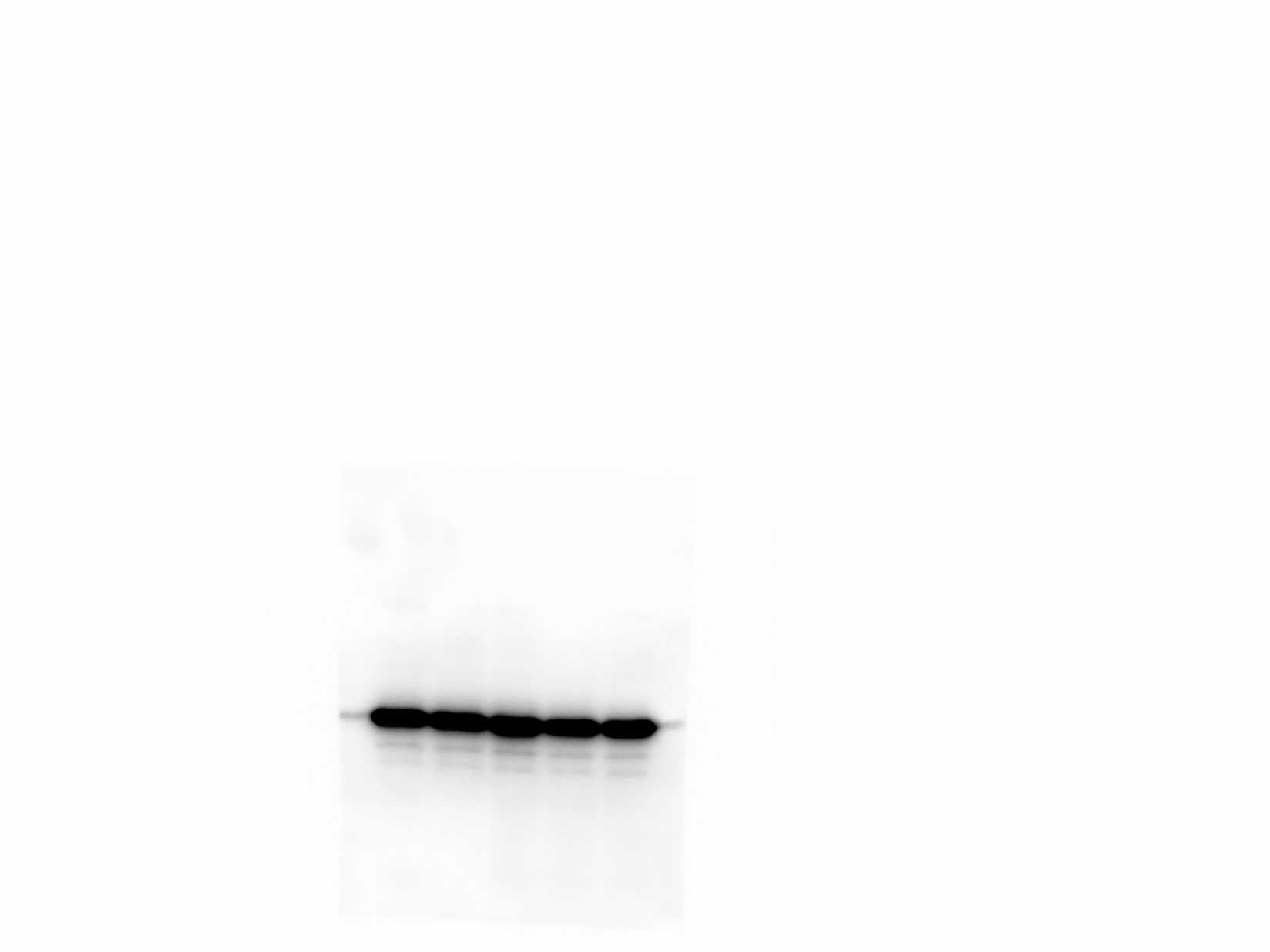

Supplement: Supplementary file 1 [file cancers-14-02406-s001.zip › Figure S7 original blots/Fig6C-gapdh.jpg]

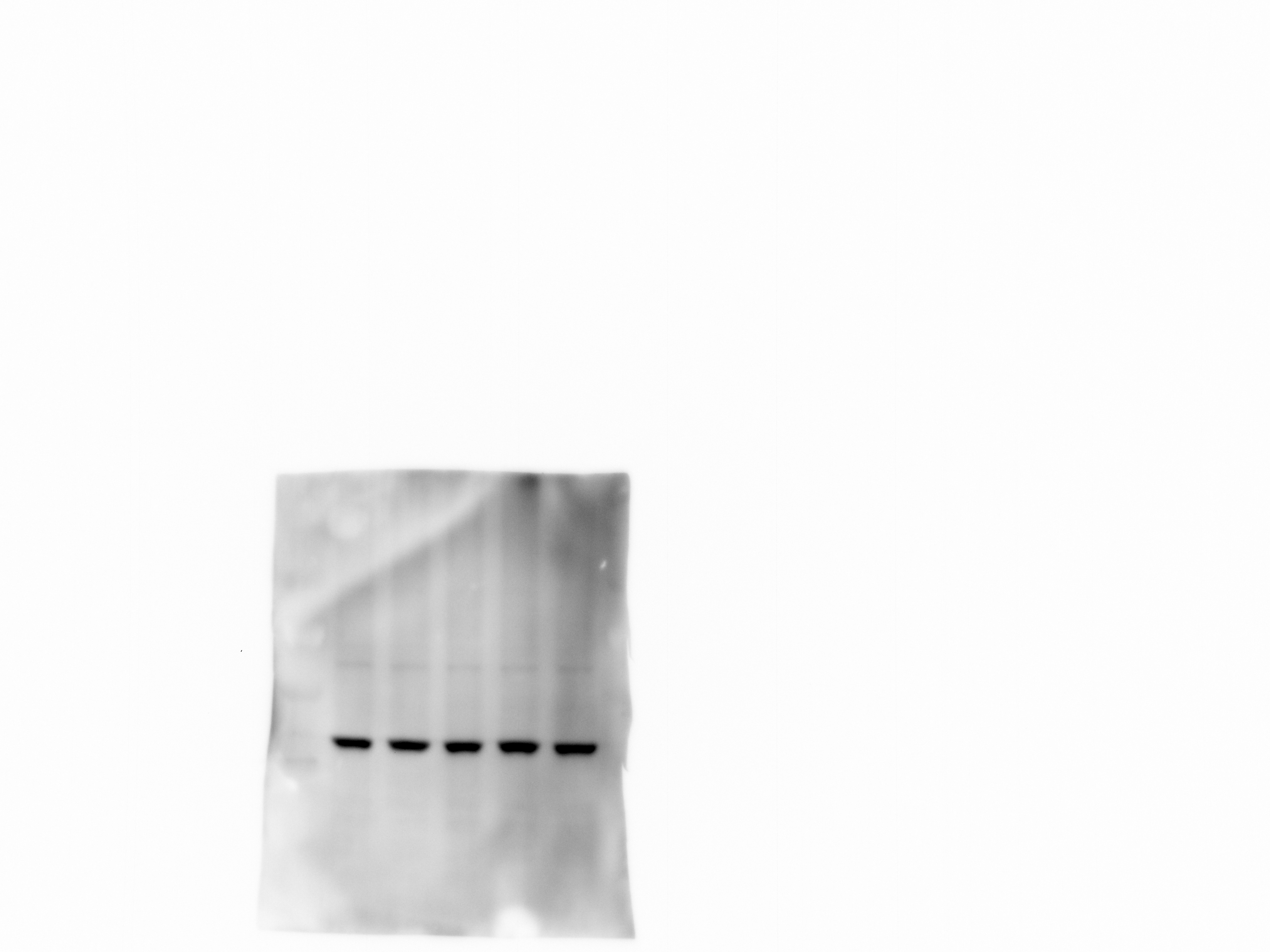

Supplement: Supplementary file 1 [file cancers-14-02406-s001.zip › Figure S7 original blots/Fig6C-p38.jpg]

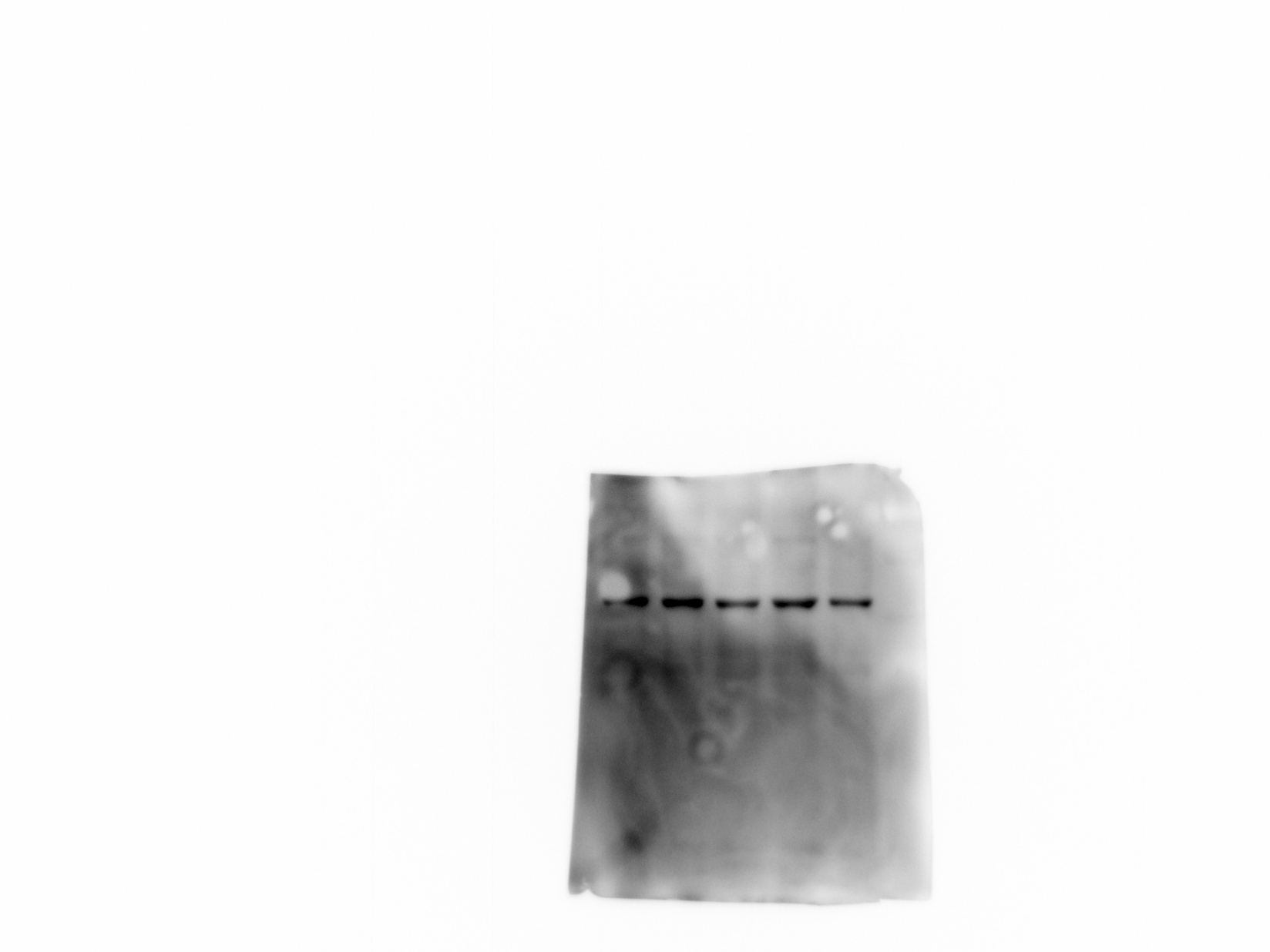

Supplement: Supplementary file 1 [file cancers-14-02406-s001.zip › Figure S7 original blots/Fig6C-pakt.jpg]

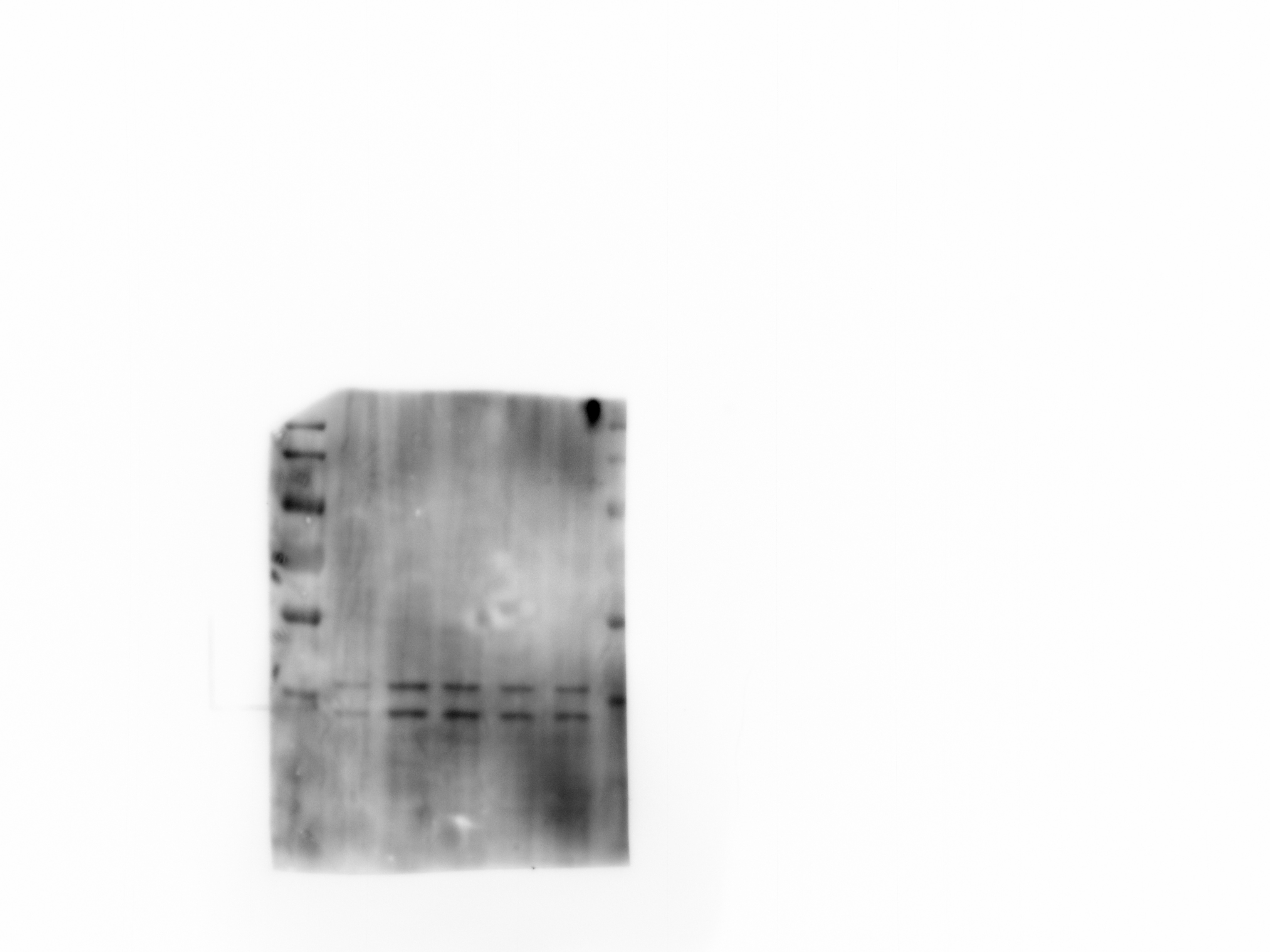

Supplement: Supplementary file 1 [file cancers-14-02406-s001.zip › Figure S7 original blots/Fig6C-perk.jpg]

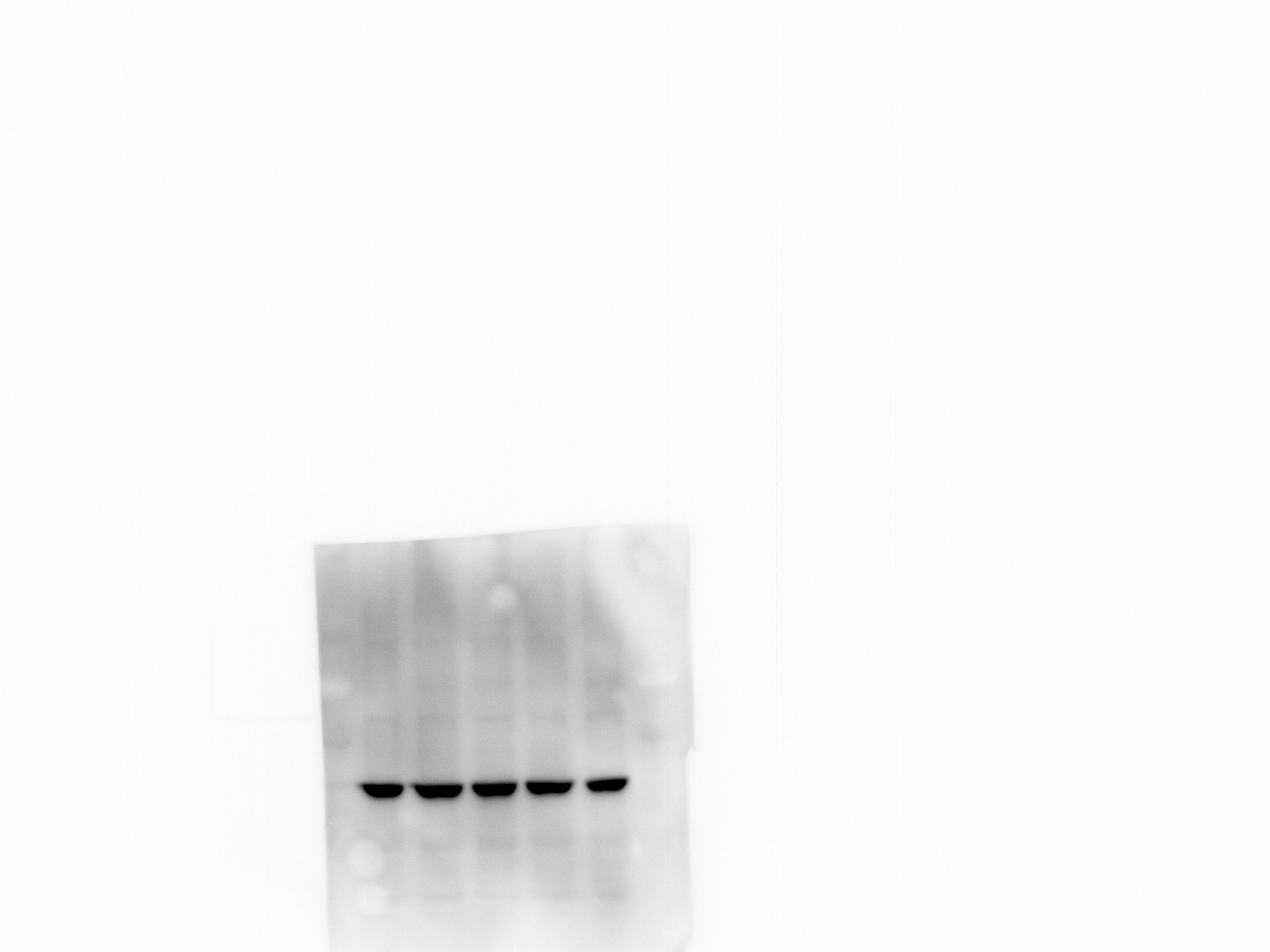

Supplement: Supplementary file 1 [file cancers-14-02406-s001.zip › Figure S7 original blots/Fig6C-pp38.jpg]

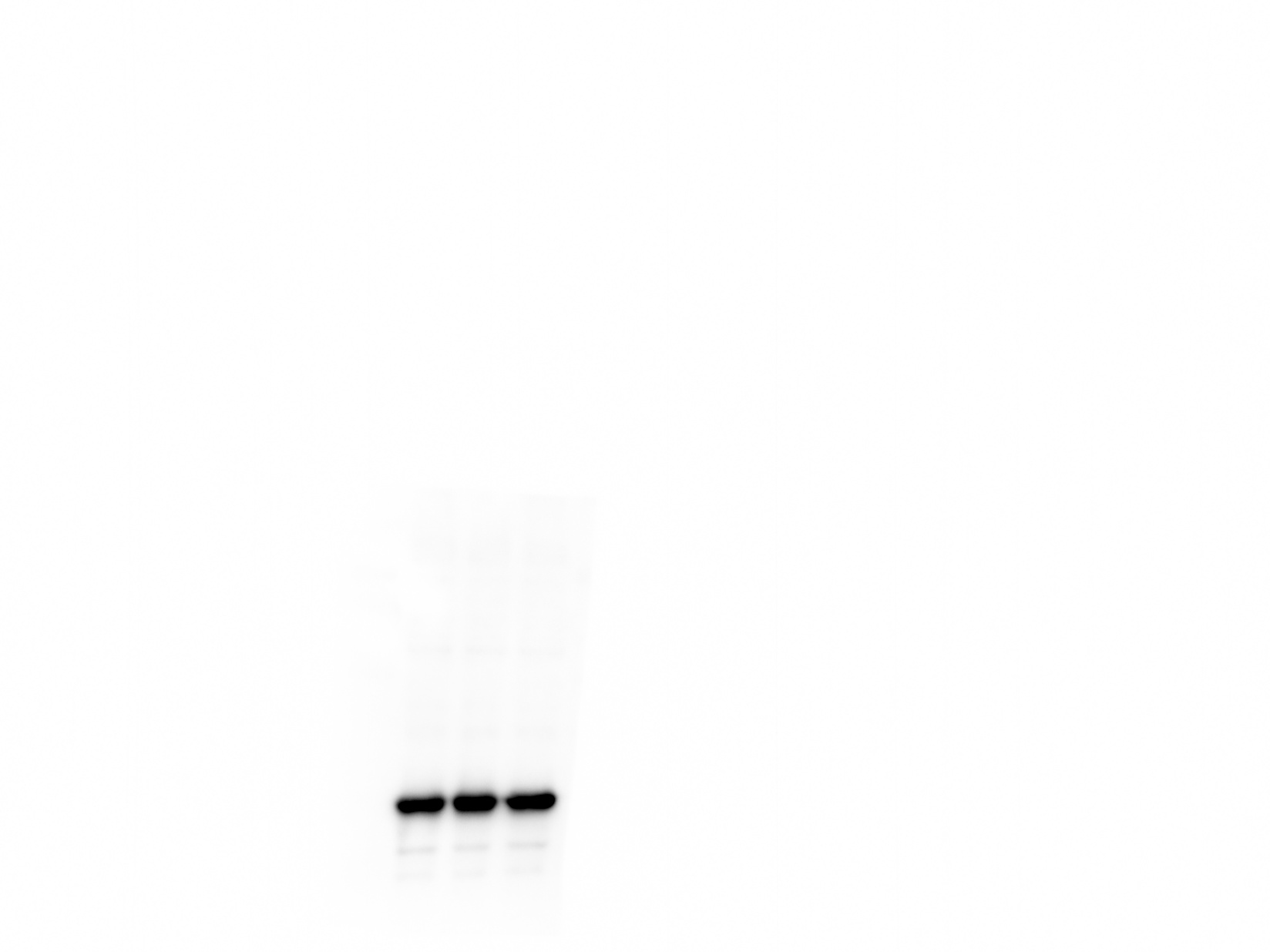

Supplement: Supplementary file 1 [file cancers-14-02406-s001.zip › Figure S7 original blots/FS3-gapdh-1.jpg]

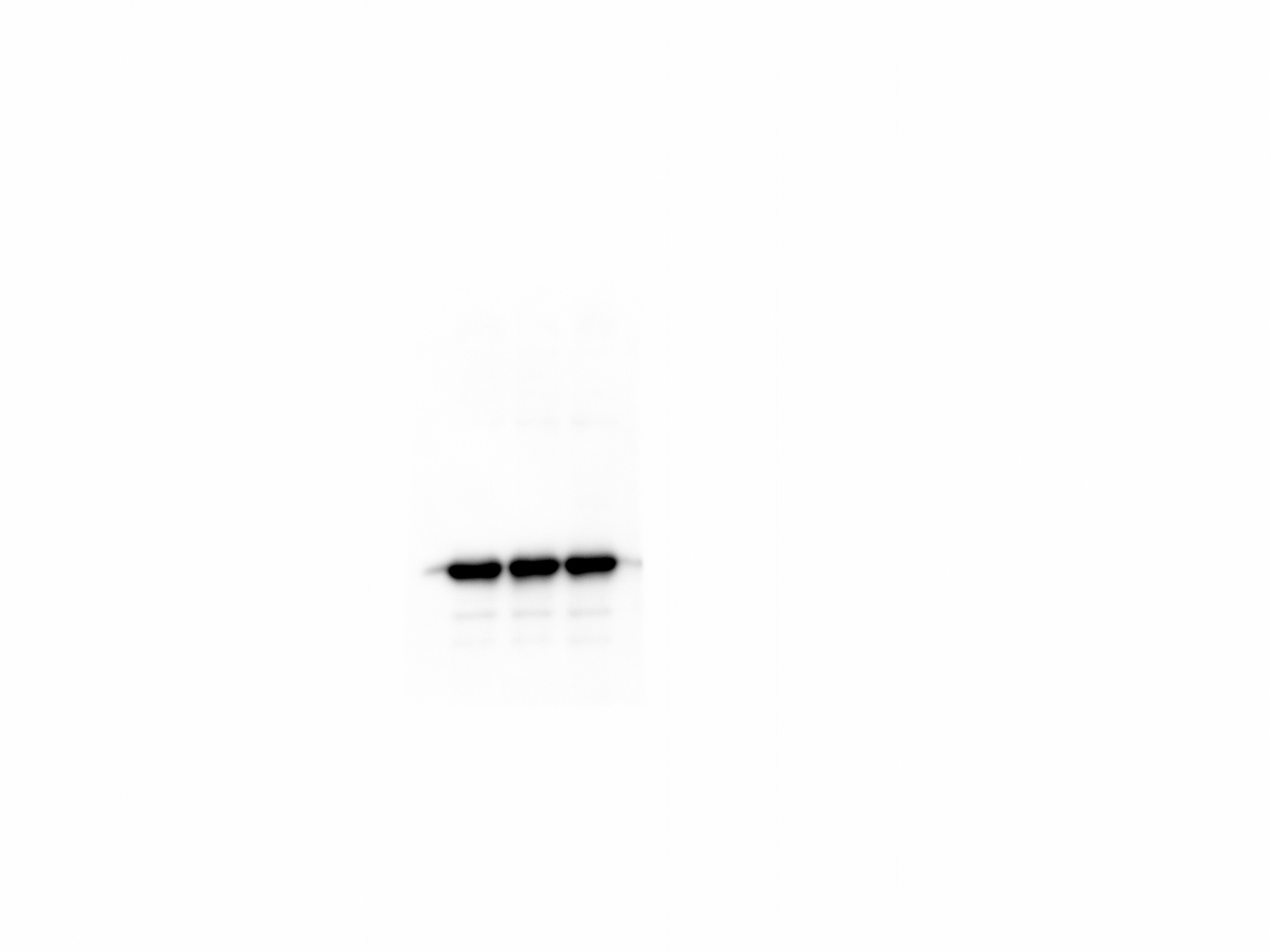

Supplement: Supplementary file 1 [file cancers-14-02406-s001.zip › Figure S7 original blots/FS3-gapdh-2.jpg]

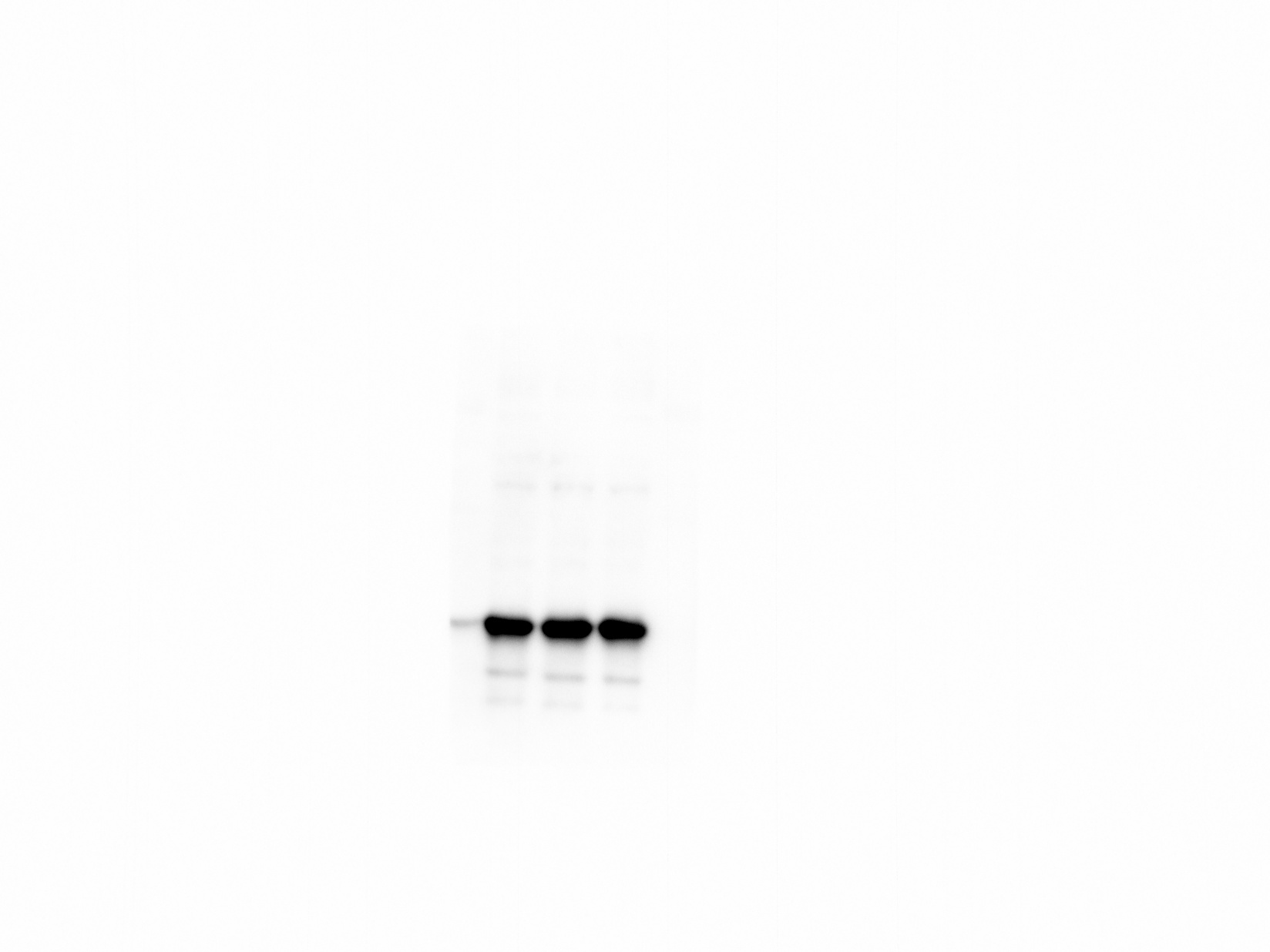

Supplement: Supplementary file 1 [file cancers-14-02406-s001.zip › Figure S7 original blots/FS3-gapdh-3.jpg]

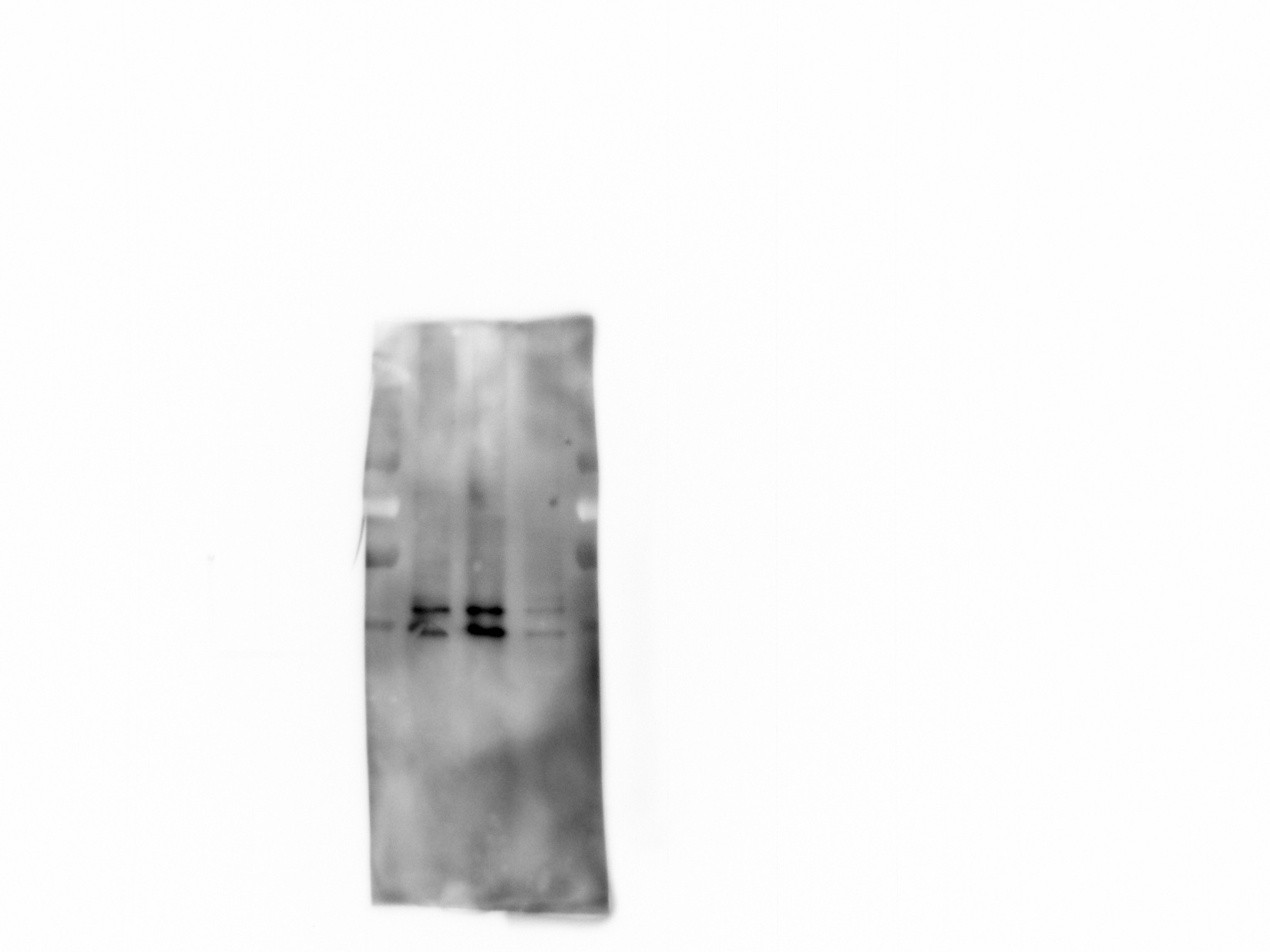

Supplement: Supplementary file 1 [file cancers-14-02406-s001.zip › Figure S7 original blots/FS3-perk.jpg]

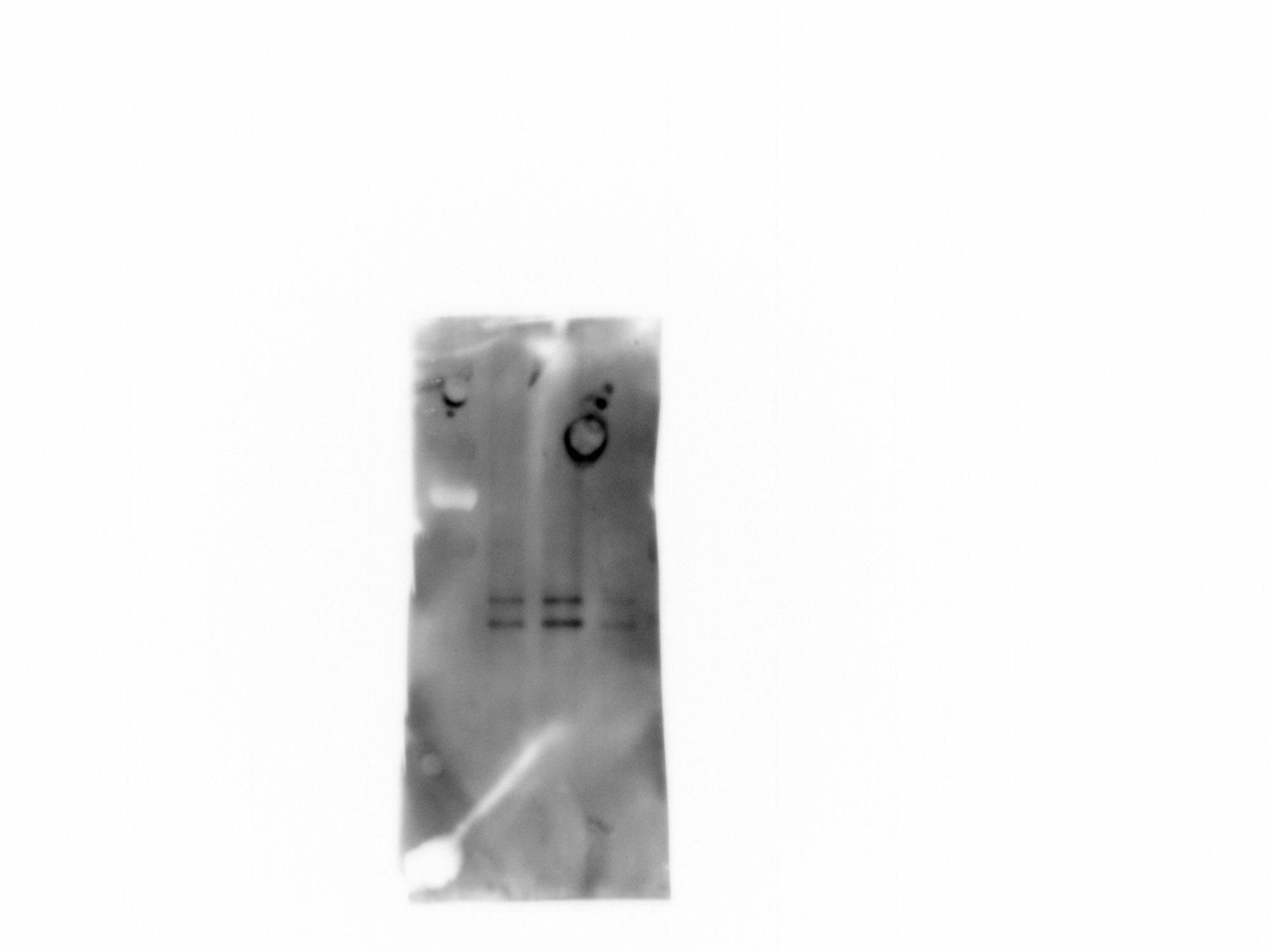

Supplement: Supplementary file 1 [file cancers-14-02406-s001.zip › Figure S7 original blots/FS3-pjnk.jpg]

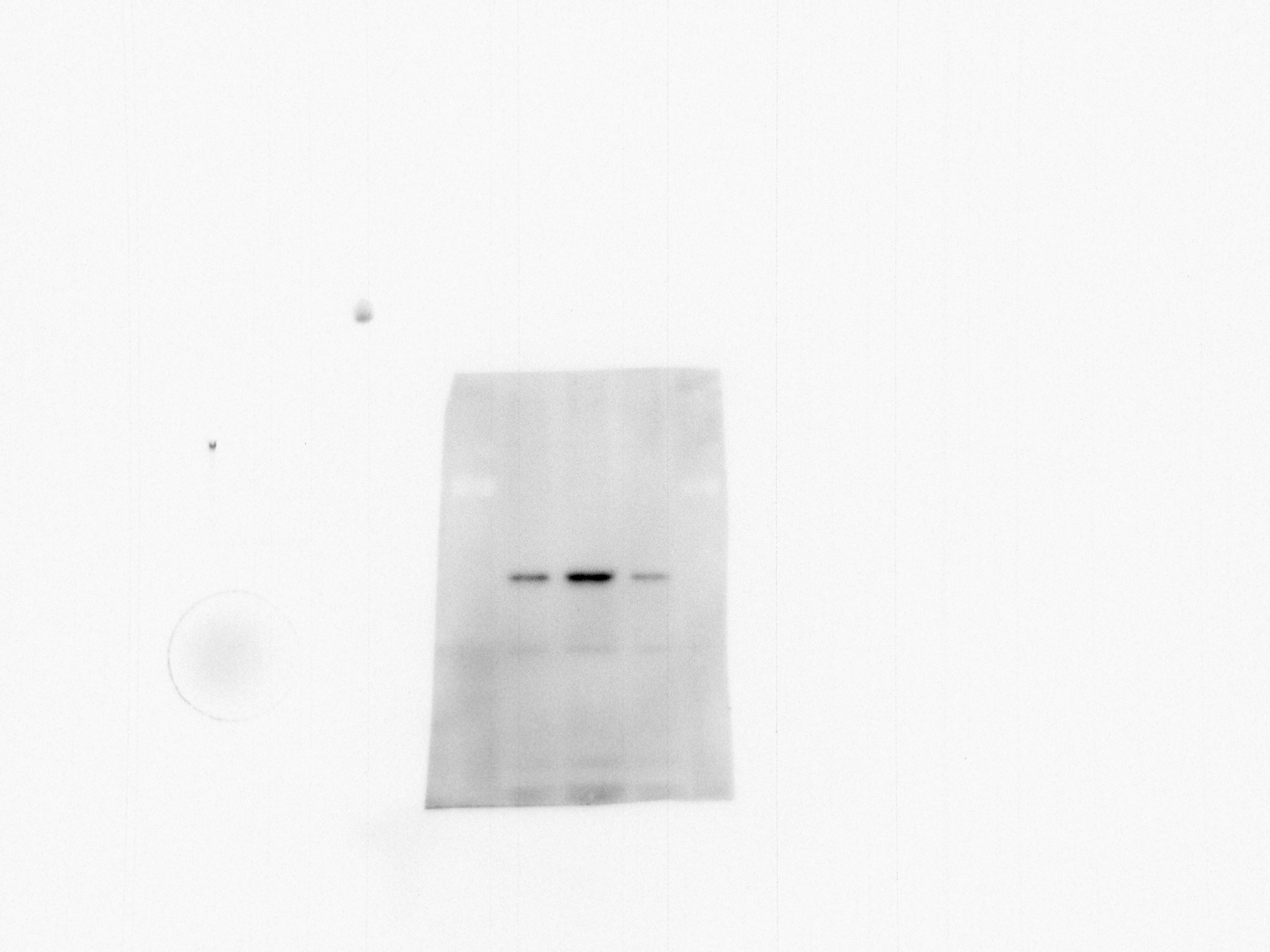

Supplement: Supplementary file 1 [file cancers-14-02406-s001.zip › Figure S7 original blots/FS3-pp38.jpg]

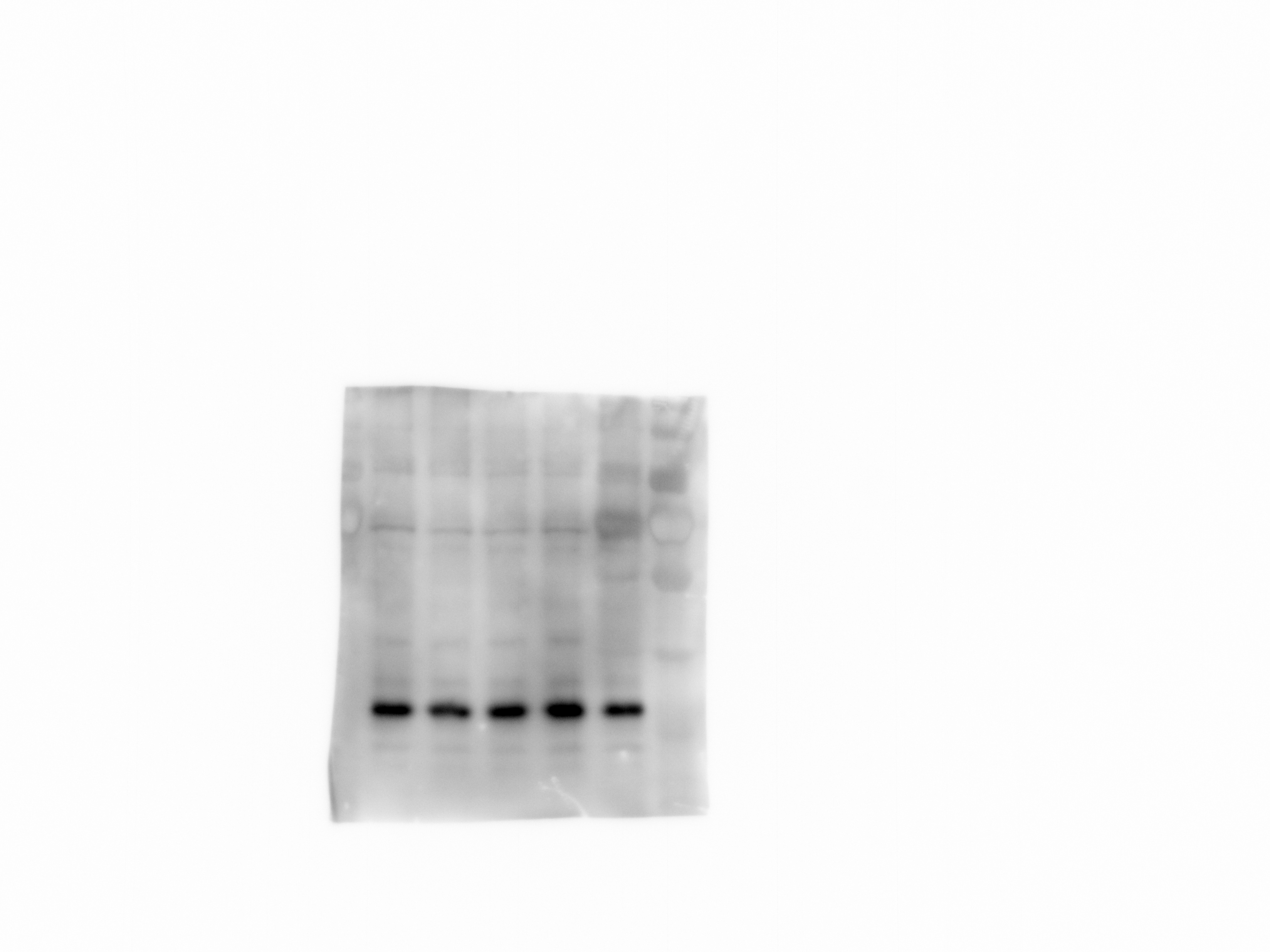

Supplement: Supplementary file 1 [file cancers-14-02406-s001.zip › Figure S7 original blots/FS3C-GAPDH.jpg]

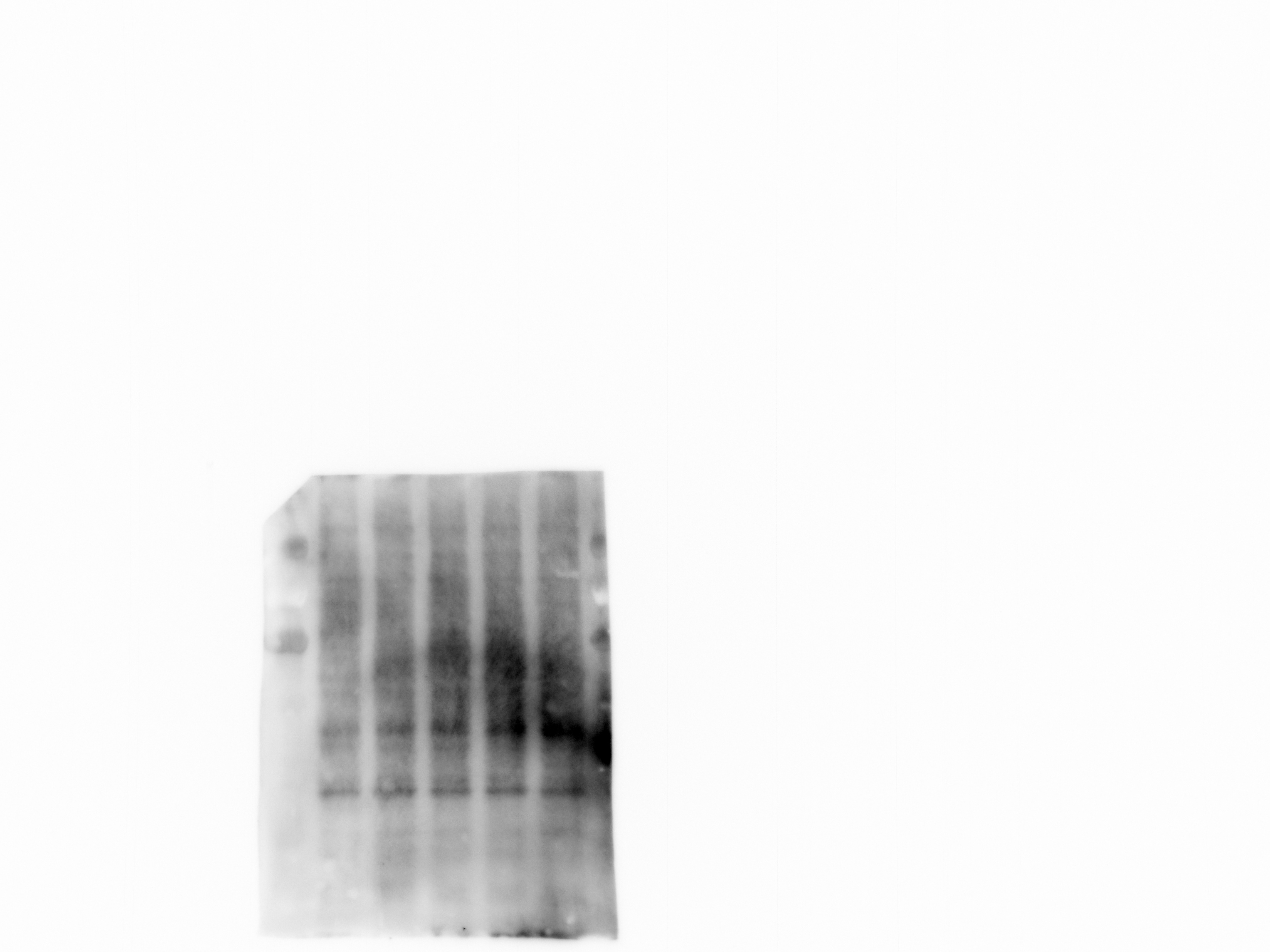

Supplement: Supplementary file 1 [file cancers-14-02406-s001.zip › Figure S7 original blots/FS3C-TLR4.jpg]

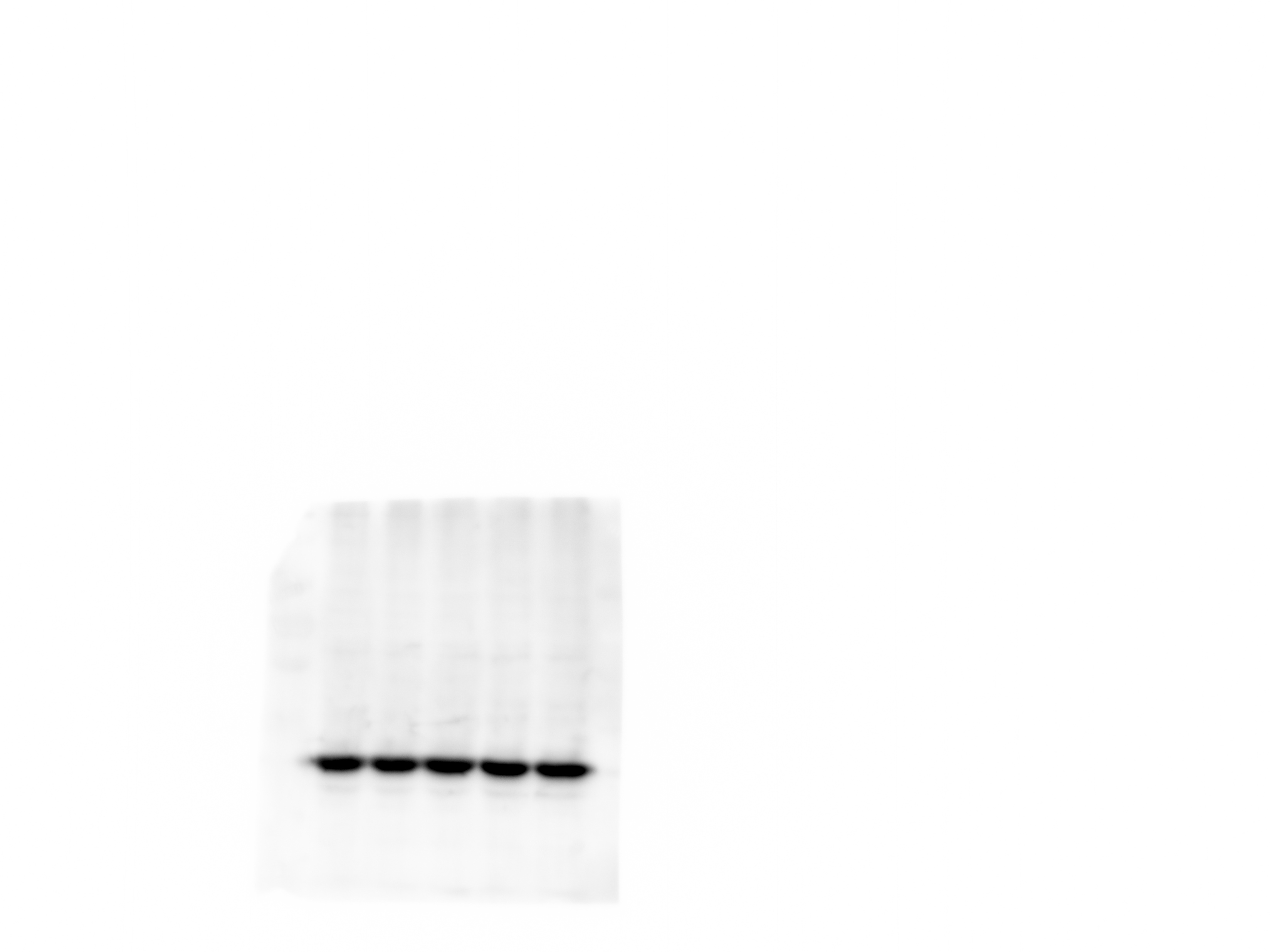

Supplement: Supplementary file 1 [file cancers-14-02406-s001.zip › Figure S7 original blots/FS5C-gapdh.jpg]

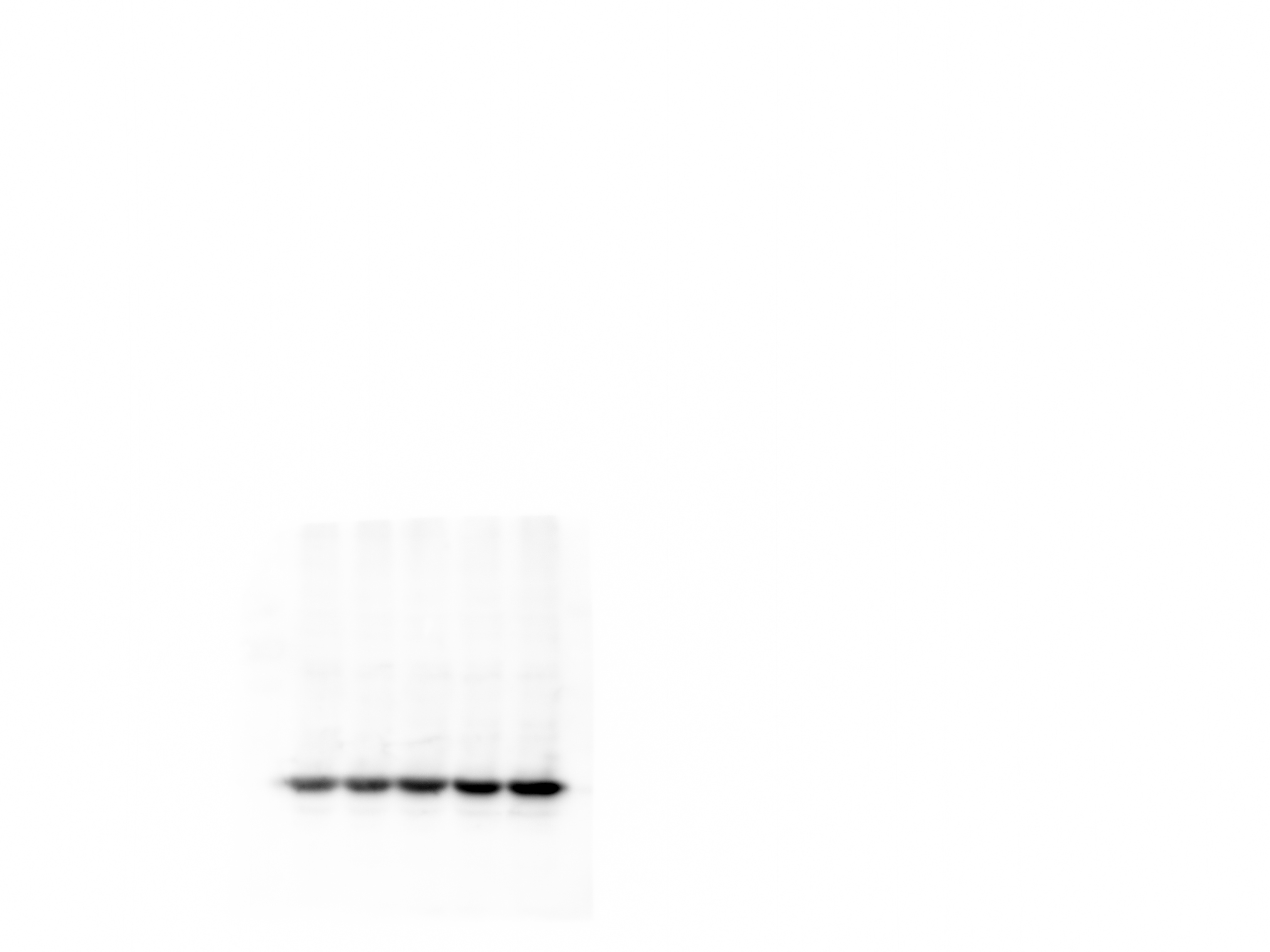

Supplement: Supplementary file 1 [file cancers-14-02406-s001.zip › Figure S7 original blots/FS5C-VEGFA.jpg]

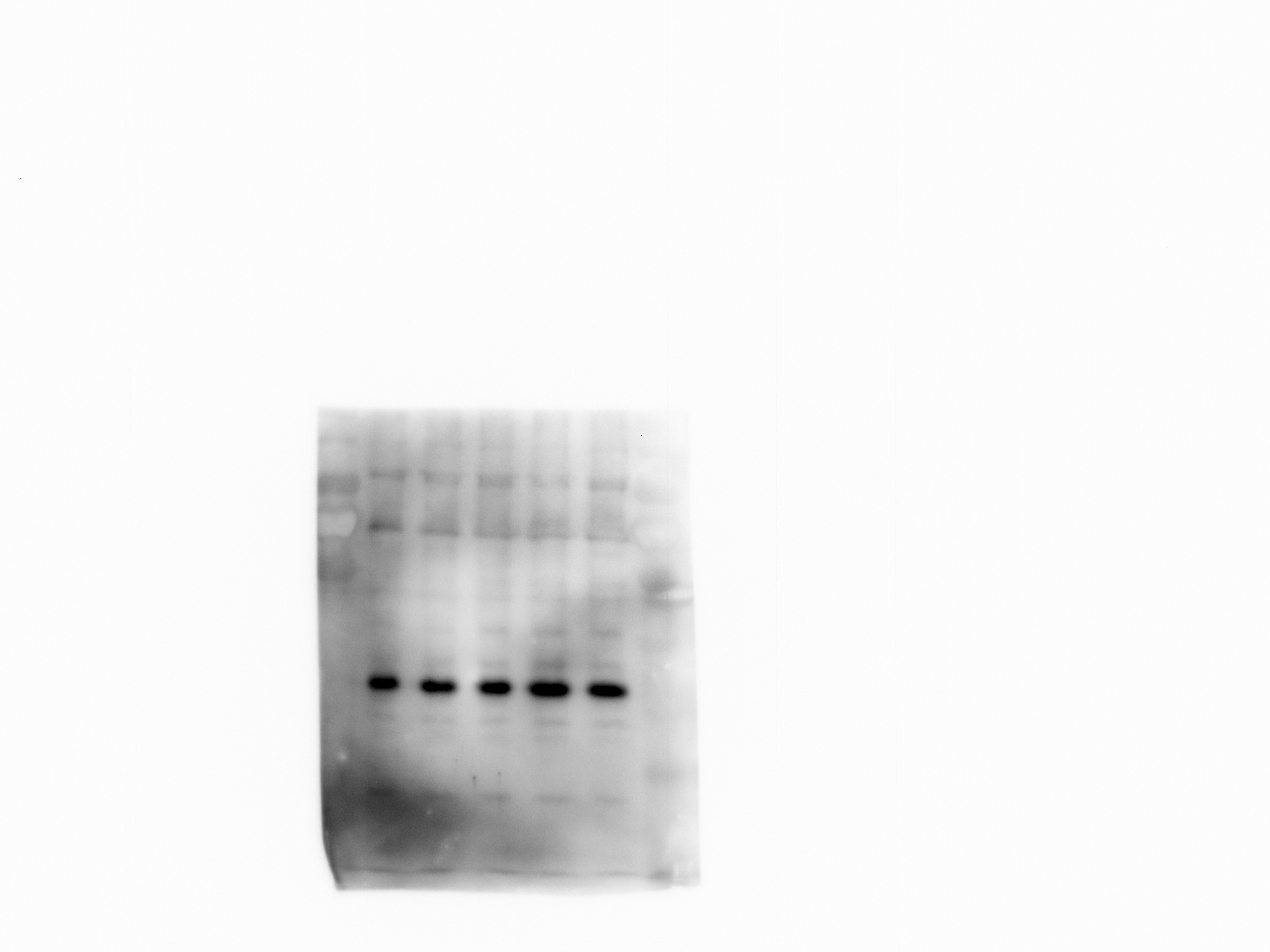

Supplement: Supplementary file 1 [file cancers-14-02406-s001.zip › Figure S7 original blots/FS5D-GAPDH.jpg]

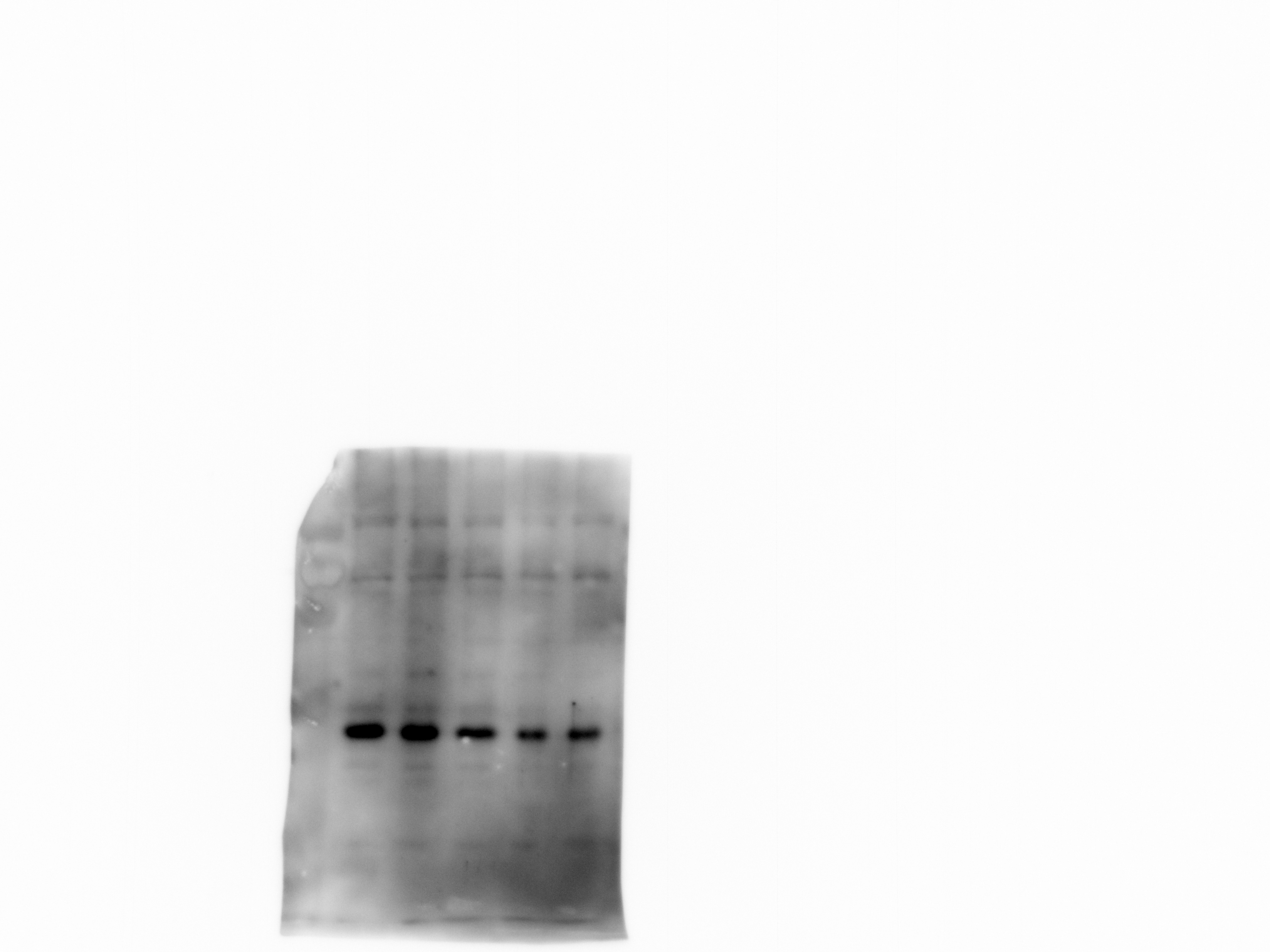

Supplement: Supplementary file 1 [file cancers-14-02406-s001.zip › Figure S7 original blots/FS5D-vegfa.jpg]
